# Identification of deubiquitinase targets of isothiocyanates using SILAC-assisted quantitative mass spectrometry

**DOI:** 10.18632/oncotarget.17261

**Published:** 2017-04-20

**Authors:** Ann P. Lawson, Daniel W. Bak, D. Alexander Shannon, Marcus J.C. Long, Tushara Vijaykumar, Runhan Yu, Farid El Oualid, Eranthie Weerapana, Lizbeth Hedstrom

**Affiliations:** ^1^ Department of Biology, Brandeis University, Waltham, MA 02453-9110, USA; ^2^ Department of Chemistry, Merkert Center, Boston College, Chestnut Hill, MA 02467-3860, USA; ^3^ Graduate Program in Biochemistry and Biophysics, Brandeis University, Waltham, MA 02453-9110, USA; ^4^ Graduate Program in Molecular and Cellular Biology, Brandeis University, Waltham, MA 02453-9110, USA; ^5^ Department of Chemistry, Brandeis University, Waltham, MA 02453-9110, USA; ^6^ UbiQ, 1098 XH Amsterdam, The Netherlands; ^7^ Current address: Department of Chemistry and Chemical Biology, Cornell University, Ithaca, NY 14853, USA; ^8^ Current address: Sanofi Genzyme, Framingham, MA 01701, USA

**Keywords:** PEITC, USP1, cisplatin, deubiquitinase, cruciferous vegetable

## Abstract

Cruciferous vegetables such as broccoli and kale have well documented chemopreventative and anticancer effects that are attributed to the presence of isothiocyanates (ITCs). ITCs modulate the levels of many oncogenic proteins, but the molecular mechanisms of ITC action are not understood. We previously reported that phenethyl isothiocyanate (PEITC) inhibits two deubiquitinases (DUBs), USP9x and UCH37. DUBs regulate many cellular processes and DUB dysregulation is linked to the pathogenesis of human diseases including cancer, neurodegeneration, and inflammation. Using SILAC assisted quantitative mass spectrometry, here we identify 9 new PEITC-DUB targets: USP1, USP3, USP10, USP11, USP16, USP22, USP40, USP48 and VCPIP1. Seven of these PEITC-sensitive DUBs have well-recognized roles in DNA repair or chromatin remodeling. PEITC both inhibits USP1 and increases its ubiquitination and degradation, thus decreasing USP1 activity by two mechanisms. The loss of USP1 activity increases the level of mono-ubiquitinated DNA clamp PCNA, impairing DNA repair. Both the inhibition/degradation of USP1 and the increase in mono-ubiquitinated PCNA are new activities for PEITC that can explain the previously recognized ability of ITCs to enhance cancer cell sensitivity to cisplatin treatment. Our work also demonstrates that PEITC reduces the mono-ubiquityl histones H2A and H2B. Understanding the mechanism of action of ITCs should facilitate their use as therapeutic agents.

## INTRODUCTION

Isothiocyanates (ITCs) are the chemoprotective natural products found in cruciferous vegetables such as broccoli, kale and watercress [1–6]. ITCs also have numerous well-documented anticancer activities, including inhibition of proliferation, induction of cell cycle arrest, apoptosis and autophagic cell death, and reduction of the inflammatory response [2, 7–9]. Phenethylisothiocyanate (PEITC) is among the best characterized ITCs due to its potent anticancer activity and low inherent toxicity. A 100 g serving of watercress releases at least 12 mg (80 μmole) of PEITC, resulting in low micromolar concentrations in human plasma [10]. Plasma concentrations of 40 μM have been observed in rats at doses used in clinical trials (100 μmole/kg) [11]. Importantly, cells can accumulate ITCs to concentrations 100-200 times those found in plasma [2].

PEITC perturbs DNA damage repair pathways [12–15] and is considered an epigenetic agent [16–19]. Moreover, PEITC sensitizes cancer cells to cisplatin treatment [12, 13, 15]. PEITC also modulates proteostasis, the inflammatory response, angiogenesis, apoptosis, cell cycle progression, proliferation and autophagy [6]. PEITC reduces the levels of critical proteins in diverse cellular pathways, including MCL1, Bcr-Abl, inhibitor of DNA binding proteins 2 and 3 (ID2 and 3) as well as apoptosis proteins such as X-IAP, cIAPs and survivin [2, 6, 9, 20–24].

**Figure 1:**
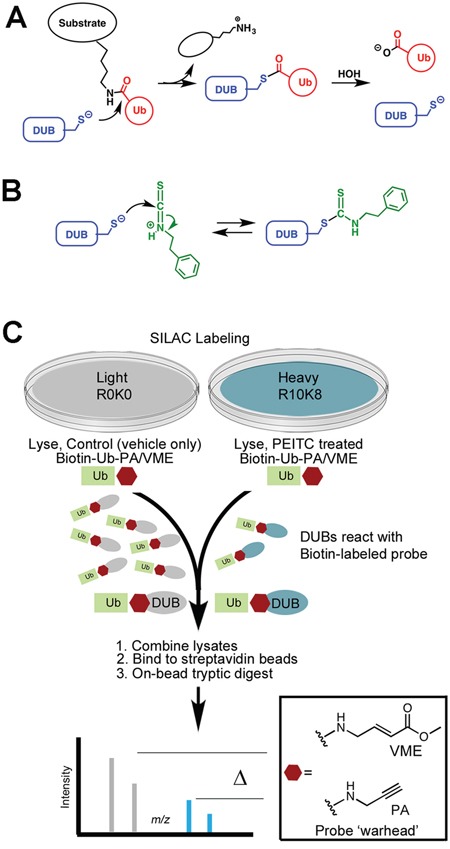
Reaction of PEITC with DUBs **(A)** Mechanism of substrate hydrolysis. **(B)** Proposed mechanism of PEITC inhibition. **(C)** SILAC-assisted quantitative proteomics strategy to identify DUBs inhibited by PEITC.

While many potential ITC targets have been identified [2–4, 8, 25], the molecular mechanisms underlying ITC activities are poorly understood. ITCs are electrophiles that form irreversible adducts with amines and reversible adducts with thiols. We previously reported that PEITC and benzylisothiocyanate (BITC) inhibit deubiquitinating enzymes (DUBs) at physiologically relevant concentrations [23]. Inhibition likely involves formation of an adduct with the active site cysteine that resembles the acylenzyme intermediate of the catalytic reaction (Figure 1A, 1B). The carbon-sulfur double bond of this dithiocarbamate adduct is longer than the corresponding carbonyl bond of the thioester intermediate, and thus is expected to capture some of the stabilizing interactions of the transition state of the DUB reaction. Two DUB targets, USP9x and UCH37, were identified, both of which are involved in cancer progression [26, 27]. The inhibition of USP9x can explain the ITC-induced knockdown of MCL1 and Bcr-Abl [21–23, 28].

Ubiquitination is a critical regulator of most cellular processes including the cell cycle, protein turnover, localization and function [29, 30], and the components of the ubiquitin (Ub) pathway are therapeutic targets for cancer and other diseases [31, 32]. DUBs regulate virtually all Ub-dependent processes [33], often protecting their protein substrates from degradation. Consequently, DUB inhibition usually reduces substrate protein levels. Therefore, DUB inhibition can explain the ITC-induced knockdown of critical oncogenic proteins. The approximately 95 DUBs encoded by the human genome are divided into 5 subclasses according to sequence similarities and likely mechanisms of action [30]. Four of these sub-families are cysteine proteases, including Ubiquitin-Specific Proteases (USP), Ubiquitin C-terminal Hydrolases (UCHs), Machado-Joseph domain-containing proteins (MJDs) and Otubain domain-containing proteases (OTUs), and thus potential targets for ITCs.

Here we employ a stable isotope labeling with amino acids in cell culture (SILAC)-assisted mass spectrometry-based (MS) activity profiling proteomics approach to more fully characterize the DUB targets of PEITC [34]. PEITC blocked the labeling of 10 DUBs, including USP1 and 6 other DUBs involved in DNA repair and chromatin remodeling. Inhibition of these DUBs provides a molecular mechanism for the ability of PEITC to sensitize cancer cells to cisplatin.

## RESULTS

### PEITC inhibits 10 DUBs

ITCs form reversible adducts with thiols, which complicates the identification of targets (Figure 1A). We previously used global cysteine activity profiling to show that PEITC does not react promiscuously with protein thiols [23]. PEITC does block the reaction of DUBs with ubiquitin affinity labels, demonstrating that DUBs are privileged ITC targets. Labeling recovered when PEITC concentrations were reduced by dilution, indicating that ITCs form reversible complexes with DUBs. USP9x and UCH37 have distinctive molecular weights that facilitated their identification as PEITC targets in these gel-based activity profiling experiments [23]. However, additional PEITC targets were clearly present in the 100 - 160 kDa molecular weight region populated by many DUBs. Therefore, we employed a SILAC-assisted mass spectrometry-based activity profiling approach to more fully characterize the DUB targets of PEITC (Figure 1C; [34, 35]). HeLa cell lysates were prepared from cells labeled with [^13^C/^15^N]-L-arginine and [^13^C/^15^N]-L-lysine (R10K8; heavy) or were unlabeled (R0K0; light). Lysates were treated with PEITC (heavy) or with 1% DMSO (light) followed by incubation with an equal mixture of biotin-ubiquitin-propargylamide and biotin-ubiquitin-vinyl methyl ester (biotin-Ub-PA/VME). These cell-free lysate experiments employed relatively high concentrations of PEITC (75 μM), in keeping with the concentrations that accumulate within cells [2, 36]. It's important to note that the reaction with the probe is time-dependent and irreversible, so high concentrations of PEITC enable the reaction to proceed long enough to label the greatest number of DUBs, yet still observe inhibition of PEITC-sensitive DUBs.

**Figure 2:**
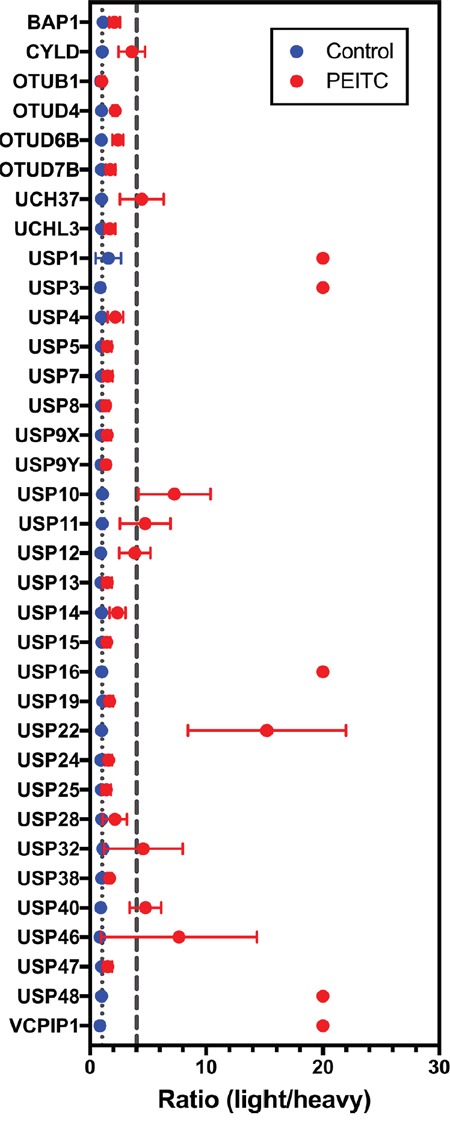
DUB targets of ITCs in HeLa cell lysates identified using SILAC-assisted quantitative MS Data presented are the mean ± standard deviation of samples from two independent biological replicates. DUBs are listed only if they appeared in both biological replicates (n = 2). The gray dotted line marks a light:heavy ratio of 1. The gray dashed line marks the threshold light:heavy ratio = 4. Peptides that are only detected in the light sample are designated an arbitrary ratio value of 20. In several samples, error bars are smaller than the symbol.

The heavy and light lysates were mixed together and the DUB-probe adducts were enriched on streptavidin beads and subjected to an on-bead tryptic digest. Two biological replicates were analyzed by quantitative MS. Light:heavy ratios were quantified for a total of 35 DUBs that were identified in both biological replicates (Figure 2, Supplementary Tables 1 and 2). PEITC inhibited the labeling of 10 DUBs as determined by a light:heavy ratio of ≥ 4 together with a statistically significant p value (p < 0.05, see Supplementary Table 1). For each of these 10 DUBs, at least 5 unique peptides were identified with consistent light:heavy ratios (Supplementary Table 2). This set included UCH37, but the inhibition of USP9x labeling was below threshold. This discrepancy with our previous report arises from a change in sample handling, specifically lysate preparation. USP9x inhibition is observed when lysates are prepared by Dounce homogenization and then treated with PEITC, as reported previously (Supplementary Figure 1A-1C and [23]). However, USP9x is resistant to PEITC when lysates are prepared by bead lysis as in the SILAC experiment. Protein activities often depend on lysis methods. Note that USP9x inhibition can be observed when cells are pretreated with PEITC prior to bead lysis and probe treatment [23].

**Table 1:** PEITC sensitive DUBs

DUB	Cellular Role/Process	Substrate(s)	PEITC
USP1	DNA repair [37],Cell cycle [37, 94]Cisplatin resistance [39]Angiogenesis	PCNA [46] ^1^FANCD2 [95]ID1 [40]ID2 [40]ID3 [40]	↓ (this work)??↓ [24]↓ [24]
USP3	Chromatin remodeling [54]DNA repair [54]Interferon signaling/antiviral immunity [96]	H2A [97]H2B [54];RIG-1 [96]	↓ (this work)↓ (this work)?
USP10	DNA repair [98]Chromatin remodeling [98]Autophagy/endocytic recycling [99]	p53 [100, 101]BECN1 [102]SNX3 [103]CFTR [104]	↓ Mutant p53 [72]???
USP11	DNA repair [98, 105]Chromatin remodeling [105]Protein stabilityTranscription [106]Inflammatory Response [107]	cIAP2 [108]I-κBα [107]PCR1 [106]γH2AX [105]	↓ [109]???
USP16	DNA repair [52, 110]Chromatin remodeling [52, 110]Cell Cycle	H2A [111]	↓ (this work)
USP22	Chromatin remodeling [79]Cell proliferation [77, 79, 112]Protein stabilityGene expression [78, 113]Telomere maintenance [114]	H2A [115]H2B [116]TRF1 [114]COX-2 [80]FBP1 [81]	↓ (this work)↓ (this work)?↓ [84]↑ p21 [82, 83, 117]^2^
USP40	?	?	?
USP48	Inflammation/immune response [73]	NF-κB/RelA [73]	↓ NF-κB gene expression [74, 75]
UCH37	Chromatin remodeling [118]Cell cycle [119]Protein homeostasis [27]	Polyubiquitin [27]	↑ K48 and K63-linked polyUb [23]
VCPIP1	Golgi Disassembly [86]	?	?

1. Abbreviations: RIG-I, retinoic acid-inducible gene 1; PCNA, Proliferating cell nuclear antigen; FANCD2, Fanconi Anemia, Complementation Group D2; H2A, histone 2A; H2B, histone H2B; PCR1, polycomb repressive complex 1; FBP1, far upstream element (FUSE)-binding protein 1; COX-2, cyclooxygenase-2; TRF1, Telomeric repeat-binding factor 1; I-κBα, nuclear factor of kappa light polypeptide gene enhancer in B-cells inhibitor, alpha; NF-κB, nuclear factor kappa-light-chain-enhancer of activated B cells; cIAP2, cellular inhibitor of apoptosis 2; BECN1, Beclin-1; SNX3, Sorting Nexin 3; CFTR, Cystic fibrosis transmembrane conductance regulator.

2. p21 is suppressed by FBP1 [81].

Intriguingly, 7 of the 10 DUBs under-represented in PEITC-treated cells are involved in DNA damage repair and chromatin remodeling (Table 1). Inhibition of these DUBs can explain the effects of PEITC on DNA repair and epigenetic regulation. USP1, USP3, USP16, USP48 and VCPIP1 were only identified in the absence of PEITC (light:heavy ratio defined as 20), suggesting that these DUBs are the most strongly inhibited. To our knowledge, the inhibition of VCPIP1 by PEITC is the first report of inhibition of an OTU domain DUB.

### PEITC decreases USP1 levels

**Figure 3:**
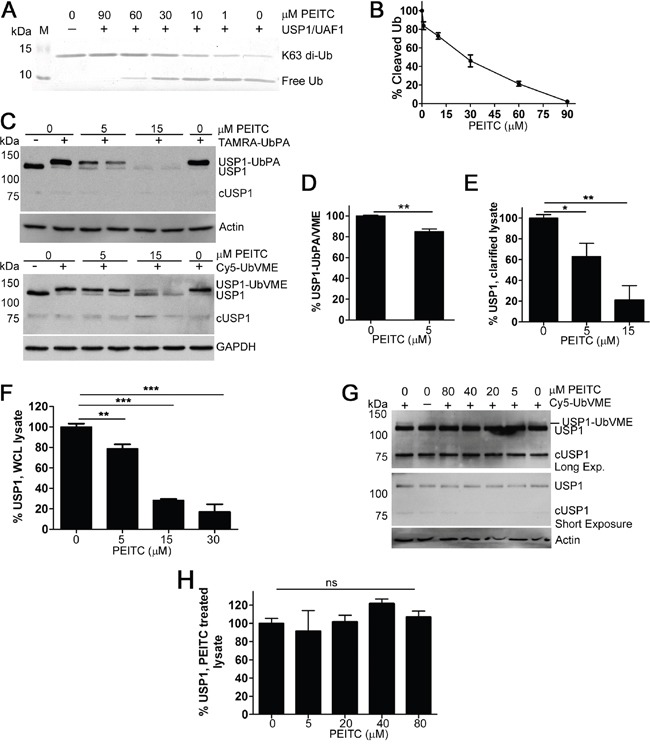
PEITC inhibits recombinant USP1 and leads to USP1 knockdown in living cells **(A)** USP1/UAF1 complex (150 nM) was pre-incubated with PEITC or with DMSO control for 8 min at which time K63-linked di-ubiquitin was added (3 μM) and incubated for 10 min at 37°C. Experiments were quenched by the addition of reducing loading buffer. Samples were analyzed by SDS-PAGE (15% gel) and the gels stained with InstantBlue (Expedeon). Data are representative of three independent experiments. **(B)** As in (A) the amount of cleaved (free) ubiquitin was measured and data were normalized to the no inhibitor control. **(C)** HEK293T cells were incubated with PEITC for 3 h, harvested, washed and lysed with glass beads. Clarified lysates adjusted to 1.6 mg/mL and treated with TAMRA-Ub-PA (1.2 μM, top) or with Cy5-Ub-VME (1.2 μM, bottom) for 8 min at 25°C and analyzed by SDS-PAGE and western blot. ‘cUSP1’ is cleaved USP1. Actin and GAPDH are shown as loading controls. **(D)** Clarified lysates were prepared as in (C) and treated with TAMRA-Ub-PA (1.2 μM) or with Cy5-Ub-VME (1.2 μM) for 8 min at 25°C. % USP1-Ub-PA/VME was determined (densitometry) by calculating the ratio of the top, USP1-probe conjugated band to total USP1 levels. Data were normalized to the DMSO (vehicle only) control. Data represent mean ± SD of 4 independent experiments. Representative blots shown in (C). **(E)** As in (C), total USP1 levels were determined by normalizing the sum of both USP1 bands to actin (or to GAPDH) and DMSO control. Data represent mean ± SD of 4 independent experiments. **(F)** HEK293T cells were incubated with PEITC for 3 h and WCLs were analyzed by SDS-PAGE and western blot. Total USP1 levels were determined by normalizing the USP1 band to the loading control (actin or GAPDH) and to DMSO control. Data represent mean ± range of at least two independent experiments. See Supplementary Figure 3A for representative blot. **(G)** A HeLa cell lysate (1.5 mg/mL) was incubated with PEITC or with DMSO (vehicle only) for 25 min at 37°C at which time Cy5-Ub-VME (1.2 μM) was added. Following a 30 min probe incubation, aliquots (equal volumes) were analyzed by immunoblotting for USP1. Actin is shown as a loading control. **(H)** As in (G) Total USP1 levels normalized to actin and are relative to the no inhibitor control. Data represent mean ± SD of 2 independent experiments. Representative blot shown in (G) ‘ns’ equals not significant. P < 0.05 *; P < 0.01 **; P < 0.001 ***

USP1 is one of the best characterized human DUBs [37]. USP1 is a critical regulator of DNA damage repair via the translesion synthesis pathway [37] and the USP1 inhibitor ML323 sensitizes cells to cisplatin [38, 39]. USP1 also deubiquitinates and stabilizes the transcriptional regulators ID1, ID2 and ID3 [40–42]. Thus the inhibition of USP1 can explain several PEITC activities: inhibition of DNA repair, sensitization to cisplatin and decrease in ID protein levels (Table 1) [13, 15, 24]. Therefore, we characterized the effects of PEITC on USP1 in more detail. We confirmed the inhibition of USP1 by PEITC by monitoring the hydrolysis of di-Ub by purified USP1 in complex with its activator UAF1 (Figure 3A, 3B). Dose dependent inhibition was observed, confirming that PEITC is a USP1 inhibitor. We also prepared lysates from HEK293T cells treated with PEITC to determine if USP1 was inhibited in living cells. Clarified lysates were treated with activity probes and USP1 was visualized by immunoblotting to observe the molecular weight shift that occurs with labeling [35, 43, 44] (Figure 3C, 3D). PEITC treatment decreased the ratio of labeled to unlabeled USP1, demonstrating that PEITC inhibits USP1 in living cells (Figure 3C, 3D and Supplementary Figure 2A, 2B). USP1 is known to undergo cleavage [45, 46] and the cleaved product, which undergoes rapid proteasomal degradation [45], was observed as a faint band with a MW of approximately 75 kDa in some, but not all, USP1 blots (Figure 3C). Surprisingly, PEITC also decreased total USP1 protein levels in a dose dependent manner (Figure 3E). The PEITC-induced knockdown of USP1 was confirmed in whole cell lysates (Figure 3F and Supplementary Figure 3A). USP1 levels were stable in cell lysates (Figure 3G, 3H), indicating that the USP1 knockdown resulted from the action of PEITC in living cells.

**Figure 4:**
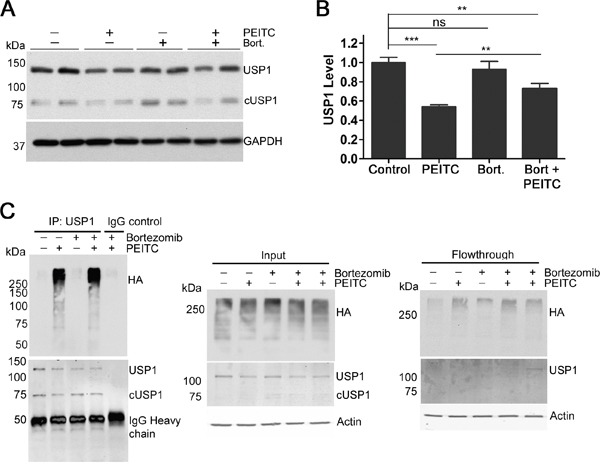
PEITC reduces levels of USP1 by increasing levels of poly-ubiquitinated USP1 **(A)** HEK293T cells were incubated with PEITC (15 μM), bortezomib (300 nM), PEITC (15 μM) together with bortezomib (300 nM) or with DMSO (vehicle only) for 3 h at 37°C. WCLs analyzed by SDS-PAGE and western blot. Data represent three independent experiments performed in duplicate. **(B)** As in (A) Data represent mean ± SD of 3 independent experiments performed in duplicate. Representative blot shown in (A). **(C)** HEK293T cells were transiently transfected with HA-Ubiquitin using TransIT2020 transfection reagent. 24 h post-transfection, cells were incubated with PEITC (15 μM), bortezomib (300 nM), PEITC (15 μM) together with bortezomib (300 nM) or with DMSO (vehicle only) for 3 h at 37°C. USP1 was immunoprecipitated and eluants were probed with anti-HA antibody and anti-USP1 antibody. Actin is shown as a loading control.

To investigate whether the PEITC-induced knockdown of USP1 was due to proteasomal degradation, HEK293T cells were incubated with PEITC in the presence of the proteasome inhibitor bortezomib (Figure 4A). Bortezomib increased total K48-linked ubiquitination as expected but had no effect on USP1 levels (Figure 4A, 4B and Supplementary Figure 3B). However, when cells were treated with PEITC in the presence of bortezomib, a significant increase in USP1 was detected when compared with PEITC treatment alone (Figure 4A, 4B and Supplementary Figure 3B). We next determined if PEITC treatment increased levels of polyubiquitinated USP1. To facilitate visualization of USP1 ubiquitination, HEK293T cells were transiently transfected with HA-tagged ubiquitin prior to treatment with PEITC, bortezomib or a combination of PEITC and bortezomib. USP1 immunoprecipitated from PEITC or PEITC/bortezomib treated lysates showed a significant amount of poly-ubiquitination (Figure 4C and Supplementary Figure 3C). In contrast, USP1 from the bortezomib or DMSO control lysates contained low or undetectable, levels of polyubiquitination. PEITC does not increase expression levels of 20S proteasomal subunits β1 or α3 (Supplementary Figure 3D, 3E), which suggests that 26S proteasome levels remain stable upon PEITC treatment. Thus PEITC induces the proteasomal degradation of USP1 by increasing the poly-ubiquitination of USP1.

### PEITC increases the ubiquitination of PCNA

**Figure 5:**
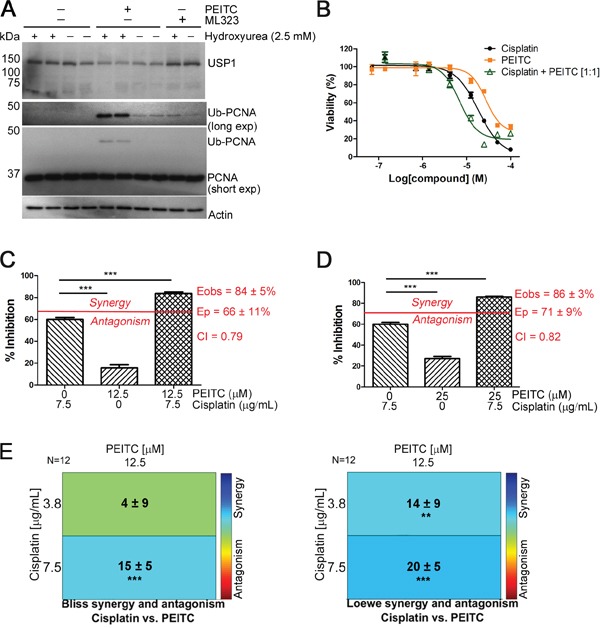
PEITC exerts a synergistic effect on the cisplatin-induced reduction of MCF-7 cell viability **(A)** HEK293T cells were incubated with PEITC (15 μM), ML323 (30 μM) or with DMSO (vehicle only) in the presence and absence of hydroxyurea (2.5 mM) for 3 h. Whole cell lysates were analyzed by SDS-PAGE and western blot. **(B)** MCF-7 cells were incubated with PEITC, cisplatin or with a 1:1 combination of PEITC and cisplatin for 48 h. Cell viability was measured using the CCK-8 assay. Data represent quadruplicate samples of at least 3 independent experiments (n ≥ 12). **(C-D)** MCF-7 cells were treated with 12 or 25 μM PEITC in combination with 7.5 μg/mL cisplatin for 48 h. Inhibition of cell growth/viability was measured using the CCK-8 assay and CI values were determined using the Bliss independence model as described in Materials and Methods. Data represent quadruplicate samples from 3 independent experiments (n = 12). **(E)**. As in (C), drug interaction was analyzed using Combenefit. Data represent quadruplicate samples from 3 independent experiments (n = 12). ‘ns’ denotes not significant. * p < 0.05; ** p < 0.01; *** p < 0.001.

USP1 regulates DNA damage repair by deubiquitinating the DNA clamp Proliferating Cell Nuclear Antigen (PCNA) [39, 46]. Treatment of HEK293T cells with PEITC for 3 h increased monoubiquityl PCNA (Ub-PCNA) both in the presence and absence of hydroxyurea, a replication inhibitor known to induce Ub-PCNA (Figure 5A). PEITC increased Ub-PCNA levels to a greater extent than the selective USP1 inhibitor ML323 (Figure 5A). The increase in Ub-PCNA is a new activity for PEITC.

### PEITC sensitizes MCF-7 cells to cisplatin

DNA damaging agents such as cisplatin are widely used in the treatment of highly aggressive, triple-negative breast cancer and BRCA1/2-mutated tumors [47–49]. Wang et al previously reported that PEITC (10 μM) potentiates the cytotoxicity of cisplatin (10 μM) in the human breast cancer line MCF-7 cells [15]. Since the concentration of PEITC used in these experiments is far greater than the highest plasma concentrations (1 μM) observed from dietary consumption [10, 50], we chose to examine the dose response of the PEITC and cisplatin interaction. Both PEITC and cisplatin caused a dose and time-dependent decreases in MCF-7 cell viability as measured using the CCK-8 assay (Figure 5B and Supplementary Figure 4A). Cisplatin and PEITC alone reduced MCF-7 cell viability with values of EC_50_ of 18 μM and 37 μM, respectively, following a 48 h drug treatment. The reduction in cell viability was potentiated when cells were treated with PEITC and cisplatin together in a 1:1 molar ratio as indicated by the reduced value of EC_50_ (6 μM) (Figure 5B). We next investigated the nature of PEITC's effect on cisplatin killing by performing Bliss independence drug interaction analysis to calculate combination index (CI) values. Synergism is indicated by a CI of less than 1, additivity by a CI equal to 1, and antagonism by a CI greater than 1. Significant synergy was observed when MCF-7 cells were co-treated with PEITC and cisplatin at concentrations of either 12 or 25 μM PEITC and 25 μM (7.5 μg/mL) cisplatin (CI values of 0.79 and 0.82, respectively; Figure 5C-5D). The interaction between cisplatin and PEITC was further analyzed using Combenefit [51] to assess three classic drug interaction models, Loewe, Bliss, and the Highest Single Agent (HSA) model. All three models showed strong synergistic effects for the combination of PEITC (12 μM) and cisplatin (12 or 25 μM), in agreement with Wang et al [15] (Figure 5E and Supplementary Figure 4B). Interestingly, combinations of PEITC and cisplatin had antagonistic effects at higher concentrations, likely reflecting the multiple pathways affected by PETIC treatment (Figure 5B and Supplementary Figure 4C-4D).

### PEITC reduces levels of mono-ubiquityl histones H2A and H2B

**Figure 6:**
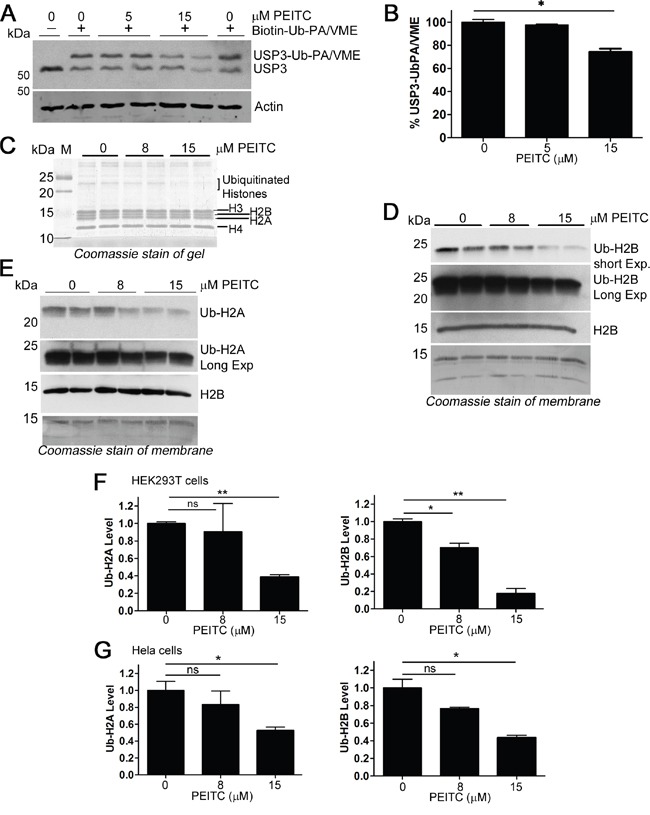
PEITC inhibits histone H2A and H2B deubiquitinases, yet reduces levels of ubiquitinated H2A and H2B **(A)** HEK293T cells were incubated with PEITC for 3 h, harvested, washed and lysed with glass beads. Clarified lysates adjusted to 1.6 mg/mL and treated with Biotin-Ub-PA/VME (1.2 μM, an equal mix of both probes) for 8 min at 25°C and analyzed by SDS-PAGE and western blot. Actin shown as loading control. **(B)** Clarified lysates were prepared as in (A) and % USP3-Ub-PA/VME was determined (densitometry) by calculating the ratio of the top, USP3-probe conjugated band to total USP3 levels. Data were normalized to the DMSO (vehicle only) control. Data represent mean ± SD of 2 independent experiments. Representative blot shown in (A) **(C-E)** HEK293T cells were incubated with PEITC or with DMSO (vehicle only) for 3 h. Cells were harvested and subjected to standard histone extraction protocol. (C) An equal volume of histone extract from each experiment was resolved on a 14% polyacrylamide gel and stained with InstantBlue (Expedeon). (D) and (E) Histone extracts (equal protein load for each lane) were analyzed by SDS-PAGE and immunoblotted with anti-ubiquityl-H2A, anti-ubiquityl-H2B, or with anti-H2B. Data are representative of two independent experiments. Coomassie stain of membrane shown to demonstrate loading. **(F)** Quantification (densitometry) of blots shown in (D) and (E), Ub-H2A and Ub-H2B protein levels were normalized to total histone levels (from Coomassie stained membrane). Data represent mean ± range of two independent experiments. **(G)** HeLa cells were incubated with PEITC or with DMSO (vehicle only) for 3h. Cells were harvested and subjected to standard histone extraction protocol. Ub-H2A and Ub-H2B protein levels were normalized to total histone levels (from Coomassie or Ponceau S stained membrane). Data represent mean ± SD of two independent experiments. Representative blots and stains shown in Supplementary Figure 5. ‘ns’ equals not significant. p < 0.05 *; p < 0.01 **; p < 0.001 ***.

Three of the PEITC-inhibited DUBs are involved in chromatin remodeling by deubiquitinating histone H2A (USP16) or both histone H2A and histone H2B (USP3 and USP22) [52, 53]. The inhibition of USP3 by PEITC in living cells was confirmed by observing the molecular shift upon probe labeling by immunoblotting (Figure 6A, 6B). USP3 depletion has been shown to increase levels of both mono-ubiquityl H2A (mono-Ub-H2A) and mono-ubiquityl H2B (mono-Ub-H2B) [52, 54]. However, contrary to expectations, treatment of HEK293T cells with PEITC decreased the levels of both mono-Ub-H2A and mono-Ub-H2B (Figure 6C-6F). Similar observations were obtained in HeLa cells (Figure 6G and Supplementary Figure 5). The broad spectrum DUB inhibitor PR-619 also decreased ubiquitylated histones [55], as do proteasome inhibitors [55, 56]. This effect has been attributed to depletion of the free ubiquitin pool due to the accumulation of high molecular weight ubiquitin conjugates [56, 57]. Importantly, decreased levels of mono-Ub-H2A and mono-Ub-H2B alter chromatin structure and disrupt DNA damage repair processes [58].

### PEITC modifies the catalytic cysteine of UCH37

**Figure 7:**
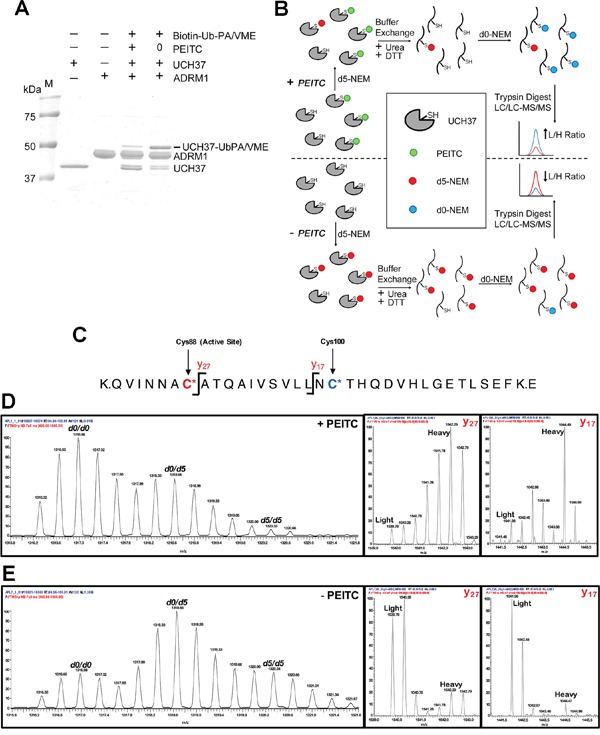
PEITC targets the catalystic cysteine in UCH37 **(A)** rUCH37/ADRM1 complex (1:1.3 molar ratio) was incubated with 1.5 mM PEITC for 15 min at 25°C and then treated with Biotin-Ub-PA/VME (an equal mix of both probes) for 5 min at 25°C. Coomassie stain of gel depicted. **(B)** Strategy for identifying PEITC labeled UCH37 cysteines. Samples (+/- PEITC) are initially treated with deuterated N-ethylmaleimide (d5-NEM), then denatured in the presence of dithiothreitol (DTT) to reverse the PEITC modification. Samples are then treated with unlabeled d0-NEM to label newly exposed cysteines and subjected to trypsin digestion. The resulting peptide samples are analyzed by LC-MS/MS. Peptides containing cysteine residues that were modified by PEITC will have a higher ratio of d0-NEM/d5-NEM labeled peptides compared to cysteine residues that did not form a PEITC adduct. **(C)** Schematic of the tryptic peptide containing the UCH37 active site cysteine residue. The active site cysteine (Cys88) is highlighted in red, while the second non-active site cysteine (Cys100) is highlighted in blue. The y17 and y27 ions fragmentation sites are also indicated. **(D)** MS1 and MS2 analysis of the C88/C100 tryptic peptide from the PEITC treated sample. Averaged d0/d0, d0/d5, and d5/d5 isotopic envelopes are displayed as well as the y17 and y27 fragmentation ions for the d0/d5 parent ion. **(E)** MS1 and MS2 analysis of the Cys88/Cys100 tryptic peptide from the untreated sample. Averaged d0/d0, d0/d5, and d5/d5 isotopic envelopes are displayed as well as the y17 and y27 fragmentation ions for the d0/d5 parent ion.

We performed several experiments to further interrogate the mechanism of PEITC inhibition of DUBs. We used UCH37, identified in both our previous report [23] and in the current SILAC experiments (Figure 2), as our model PEITC-sensitive DUB because it is reasonably well characterized, its small size is more amenable to peptide identification and suitable quantities could be obtained by recombinant protein expression. UCH37 is activated by the proteasomal subunit ADRM1 (also known as RPN13) [59]. PEITC inhibits rUCH37/ADRM1 complex binding to active site directed probe, TAMRA-Ub-PA (Figure 7A) and inhibits UCH37 in living cells (Supplementary Figure 2C, 2D) [23]. The preincubation of rUCH37/ADRM1 with PEITC also inhibited the hydrolysis of Ub-Rho110MP (Supplementary Figure 6). No rUCH37/ADRM1 activity recovered when DTT was added after preincubation, indicating that the PEITC adduct with rUCH37/ADRM1 is stable (Supplementary Figure 6). Unfortunately, UCH37/ADRM1 inactivates under these assay conditions, so we can only conclude that the PEITC adduct does not react rapidly with dithiothreitol on the folded enzyme. Nonetheless, the stability of this adduct suggested that it might be possible to use MS analysis to determine if the catalytic Cys88 was modified.

Direct detection of the proposed PEITC adduct with the catalytic Cys88 of UCH37 was not feasible by MS analysis due to the instability of this adduct to protein denaturation and trypsin digestion, which must be performed in the presence of reducing thiols to prevent disulfide bond formation. Instead, we sought to quantify PEITC occupancy of the active-site Cys88 using differential isotopic labeling of cysteines in the presence and absence of PEITC. Briefly, UCH37 samples (+/-PEITC) were treated with isotopically heavy *N*-ethyl maleimide (d5-NEM) to cap all exposed and reactive cysteine residues (Figure 7B). After UCH37 denaturation and treatment with DTT, any newly exposed cysteines were then capped with isotopically light NEM (d0-NEM). High-resolution MS analysis was used to quantify the light versus heavy intensities for each cysteine-containing peptide. A cysteine targeted by PEITC would be expected to display an increase in the d0-species and a decrease in the d5-species relative to the untreated sample. However, analysis of the active-site Cys88 was complicated by the presence of a second cysteine residue (Cys100) on the same peptide, affording three potential NEM adducts (d5/d5, d0/d5 and d0/d0) (Figure 7C). An increase in the d0/d5 and the d0/d0 species in the +PEITC sample is observed relative to the –PEITC sample, indicating that PEITC targets one or both of these two cysteine residues (Figure 7D and 7E). To assign the site of d0-NEM labeling, the fragmentation (MS/MS) spectra were analyzed for fragments that contained d0 versus d5 NEM. Specifically, for the two fragment ions that contain Cys100 but not Cys88 (y17 and y27), the predominant species in the +PEITC sample contains the d5-NEM labeling on Cys100 (Figure 7D and 7E). Therefore, treatment with PEITC results in an increase in d0-NEM labeling at Cys88, indicating that the active-site cysteine is the site of PEITC modification.

## DISCUSSION

Dietary ITCs such as PEITC have well-established anticancer activities and numerous potential targets of ITCs have been identified [2–4, 6]. Nonetheless, the molecular mechanisms of ITC action are poorly understood. Clearly understanding the molecular basis of ITC action is important for identifying the diseases that would most benefit from treatment.

DUBs regulate processes considered critical hallmarks of cancer such as cell proliferation, angiogenesis and apoptosis. Our work demonstrates that PEITC is a broad spectrum DUB inhibitor at physiologically relevant concentrations [23]. We previously identified two DUB targets of PEITC, UCH37 and USP9x. The inhibition of USP9x accounts for the PEITC-induced decreases in the oncoproteins MCL1 and Bcr-Abl. Here we identify 9 additional PEITC-sensitive DUBs, which can explain many cellular effects of PEITC (Table 1). Chief among these is the ability of PEITC to sensitize cancer cells to cisplatin, and reverse cisplatin resistance [12–15]. Cisplatin crosslinks DNA, and mechanisms of resistance include increased DNA repair as well as enhanced tolerance of DNA damage [60]. Seven PEITC-sensitive DUBs have well-recognized roles in DNA repair or chromatin remodeling, including USP1, USP3, USP10, USP11, USP16, USP22 and UCH37. This polypharmacology is in keeping with the complicated relationship between cisplatin and PEITC action observed in our experiments, with synergism observed at low concentrations and antagonism observed at high concentrations. The synergistic interaction, at least in MCF-7 cells under the conditions of our experiments, requires higher concentrations of PEITC than obtained from dietary consumption, but is in the range of the concentrations used in drug trials.

USP1 has garnered increasing attention as an attractive therapeutic target [38, 39, 41, 42]. USP1 expression is upregulated in several cancers [37] and its dysregulation has been linked to cisplatin-resistance both in non-small cell lung cancer and osteosarcoma cells [39, 61]. USP1 over-expression is associated with cancer aggressiveness in multiple tumor types [40, 62, 63]. The USP1-selective inhibitor ML323 sensitizes cancer cells to cisplatin, demonstrating that USP1 inhibition is sufficient to explain the effects of PETIC. PEITC is more effective than ML323 as judged by accumulation of Ub-PCNA, possibly because it both inhibits USP1 and decreases USP1 levels (note that both the accumulation of Ub-PCNA and the knockdown of USP1 are also new activities for PEITC). Our experiments indicate that PEITC increases the ubiquitination and subsequent degradation of USP1 by the proteasome. USP1 is targeted for proteasomal degradation by the multi-subunit E3 ligase, APC/C^Cdh1^ in a cell cycle dependent manner [64], so perhaps the decrease in USP1 is a consequence of the ability of PEITC to cause cell cycle arrest [2, 65]. Alternatively, USP1 may be protected from degradation by another PEITC-sensitive DUB.

While USP1 inhibition is sufficient to account for the ability of PEITC to sensitize cells to cisplatin, the other inhibited DUBs are also likely to play important roles. The decrease in mono-Ub-H2A and -H2B, another new PEITC activity revealed in the current work, will also promote cisplatin sensitivity. Both histones play important roles in the epigenetic control of gene expression, cell cycle progression, and DNA damage repair [33, 66], functions that are all perturbed by PEITC treatment. Histone deubiquitination alters chromatin structure and limits access of DNA repair proteins to damaged sites, thus sensitizing cells to cisplatin [58]. Proteasome inhibitors also reduce mono-Ub-H2A and -H2B and sensitize cells to cisplatin [58, 67], as does another broad-spectrum DUB inhibitor [55]. All of these agents cause the accumulation of poly-ubiquitinated proteins, and the consequent depletion of free Ub causes a re-distribution of Ub from the nucleus to the cytosol that results in the de-ubiquitination of histones H2A and H2B [67].

Our SILAC experiments also provide possible explanations for other previously described PEITC activities and suggest new PEITC activities. The PEITC-induced decrease in ID2 and ID3 can be attributed to the inhibition/knockdown of USP1 [24, 40]. ID proteins are transcriptional regulators required for the maintenance of cancer stem cells. In addition, ID proteins are implicated in tumor angiogenesis and induce expression of pro-angiogenic factors including hypoxia-inducible factor-1α (HIF1α), vascular endothelial growth factor A (VEGFA), interleukin-6 (IL-6) [40, 68]. Interestingly, PEITC treatment lowers levels of each of these angiogenic proteins [69–71]. The ability of PEITC to reduce the levels of mutant p53 levels may be attributed to the inhibition of USP10 [72]. Moreover, while the cellular functions of USP48 are not well defined, it controls the proteosome-dependent turnover of activated NF-κB/RelA in the nucleus, and thus regulates inflammatory and immune response. Depletion of USP48 decreases NF-κB target gene expression [73]. Thus inhibition of USP48 can explain how PEITC reduces the inflammatory response and the associated markers iNOS, TNF-α and IL-6 [74, 75]. USP22 is a member of the 11-gene ‘death-from-cancer’ signature that predicts cancer aggressiveness and the likelihood of treatment failure in a wide range of malignancies [76–79]. USP22 is a subunit of the human SAGA (hSAGA) transcriptional regulation complex and contributes to cancer ‘stemness’ by activating a range of genes. USP22 deubiquitinates other substrates; it stabilizes COX-2 [80] and it trims K63-linked polyubiquitin from transcriptional regulator FBP1 [81], a requirement for FBP1-mediated repression of the cell-cycle inhibitor p21^WAF1^. Thus inhibition of USP22 can explain the mechanisms underlying PEITCs ability to both increase p21^WAF1^ levels [82, 83] and decrease levels of COX-2 [71, 84]. VCPIP1 (also known as VCIP135) has specificity for K11 and K48 linkages and is involved in p97/p47-mediated processes [85]. VCPIP1 is required for the reassembly of Golgi stacks after mitosis [86]. These observations suggest that PEITC will also perturb these processes. Lastly, PEITC-downregulated proteins are attractive substrate candidates for the uncharacterized DUB USP40. It is very likely that the promiscuous inhibition of DUBs, and the resulting pleiotropic effects, is an important factor in the anticancer activity of PEITC. Indeed, the importance of such polypharmacology in drug efficacy is increasingly recognized [87].

Cruciferous vegetables and dietary ITCs are in more than a dozen clinical trials for various cancers including lung, oral, prostate, melanoma, breast, and pancreatic. Trials are also underway to investigate the effects of ITCs on inflammation and the immune response. Understanding the mechanism of action of ITCs should facilitate their use in the clinic and may also lead to the discovery of novel DUB-substrate interactions, new therapeutic targets, and the development of more potent and selective DUB inhibitors.

## MATERIALS AND METHODS

### Reagents

All chemicals and reagents were from Sigma Aldrich unless otherwise stated. Solvents (except DMSO) were from Fisher (Pittsburg, PA). Other reagents used in this study: PEITC (Acros Organics); Bortezomib (Millennium Pharmaceuticals); Mini-Complete and PhosSTOP inhibitory cocktails (Roche Applied Science); recombinant human K63-linked di-ubiquitin (UC-300), recombinant human His_6_-USP1/UAF1 complex (E-568; Boston Biochem.); DMEM, glutamax, penicillin/streptomycin (Gibco); trypsin (0.25%), DPBS (Hyclone); cell dissociation buffer (fisher); Bradford dye (Bio-rad); dithiothreitol (GoldBio Tech); PVDF hybond, Amersham ECL Prime WB detection reagent (GE Healthcare Life Sciences); Blue Biofilm (Denville Scientific); ML323 USP1 inhibitor (EMD Millipore); TransIT 2020 transfection reagent (Mirus bio); Pierce protein G magnetic beads (ThermoFisher Scientific); TAMRA-ubiquitin propargylamide (TAMRA-Ub-PA), Cy5-ubiquitin vinyl methyl ester (Cy5-Ub-VME), Biotin-Ahx-Ub-VME, Biotin-Ahx-Ub-PA and Ub-Rh110MP (UbiQ). HA-ubiquitin vinylsulfone (HA-Ub-VS) and HA-Ub-VME were synthesized using standard methods previously described [88]. The plasmid encoding the HA-Ub(1-75)-intein-chitin binding domain fusion protein was a gift from Prof H. Ploegh of the Whitehead Institute.

### Antibodies

The following antibodies were used: anti-K48-linked ubiquitin, clone APU2 (Millipore); anti-PARP 9542, anti-USP1 D37B4, anti-ubiquityl-Histone H2A (Lys119), anti-ubiquityl-histone H2B (Lys 120), anti-histone H2B, goat anti-rabbit IgG (HRP), anti-ADRM1, normal rabbit IgG (Cell Signaling Technologies); anti-actin clone AC-40 A3853, anti-GAPDH clone G9295, (Abcam); anti-USP3 (rabbit polyclonal) (GeneTex); anti-pcna PC10, anti-Proteasome α3 clone A-9, anti-Proteasome β1 clone D-9, anti-Nrf2 clone A-10 (Santa Cruz); Cy3 conjugated secondary antibodies (GE Life Sciences); anti-UCH37, goat anti-mouse IgG (HRP) (Abcam). IRDye 800CW donkey anti-rabbit and IRDye680CW donkey anti-mouse were obtained from Li-Cor.

### Vehicle

All compounds were dissolved in DMSO and further diluted with culture medium before use for tissue culture assays (final DMSO concentrations were less than 0.1%). For *in vitro* assays, the final DMSO concentration was 1%.

### Tissue culture assays and western blot

HeLa (ATCC, purchased June, 2015), MCF-7 and HEK293T cells (ATCC, each authenticated June 2015, 9-marker STR, IDEXX BioResearch) were cultivated in DMEM supplemented with 10% heat inactivated FBS, 1X glutamax, and 1X penicillin/streptomycin under standard conditions (37 °C in a 5% CO_2_ humidified atmosphere). All active cell cultures routinely tested for presence of Mycoplasma (MycoAlert^TM^ detection kit, Lonza) and confirmed to be Mycoplasma free.

Whole cells lysates (WCLs) for immunoblots were prepared according standard protocol. Protein concentration was determined using Bradford assay with IgG as standard. For acid extraction of histones, cells were lysed in TEB buffer (phosphate buffered saline containing 0.5% Triton X-100) containing 1x protease inhibitor cocktail and insoluble pellets were resuspended in 0.2 M HCl and incubated overnight at 4°C; the acid was neutralized with 1 M Tris-HCl (pH 8.5) for Western blot analysis. Proteins were resolved by SDS-PAGE, transferred onto PVDF membranes and immunoblotted with the appropriate antibody. Signals were visualized with HRP-conjugated secondary antibodies and ECL Prime reagent (Amersham GE). In certain cases, signals were visualized with Cy3-conjugated secondary antibodies (scanned on GE Typhoon scanner) or with IRDye800CW or IRDye680CW (scanned on Licor scanner). Densitometry was performed with ImageJ software (http://rsbweb.nih.gov/ij/download.html).

### PEITC inhibitor selectivity as determined by quantitative mass spectrometry

HeLa cells were grown in DMEM media minus L-lysine and L-arginine (Thermo Scientific Pierce) supplemented with 10% dialyzed FBS for SILAC (Pierce), 1x glutaMax (Gibco), 1% penicillin/streptomycin (Gibco) and either 84 μg/mL [13C/15N]-L-arginine (R10) and 146 μg/mL [^13^C/^15^N]-L-Lysine (K8) (Cambridge Isotope Laboratories) or 84 μg/mL L-arginine (R0) and 146 μg/mL L-lysine (K0) (Sigma) at 37°C and 5% CO_2_ for a minimum of 6 passages (complete metabolic labeling of proteome confirmed by MS). Heavy labeled or unlabeled HeLa cells were lysed with glass beads in DUB buffer and protein concentration adjusted to 1.4 mg/mL. Heavy lysates were incubated with PEITC (75 μM) and light lysates with DMSO (vehicle only, 1%) for 25 min at 37°C at which time Biotin-Ahx-Ub-VME/PA (equal mix of both biotin probes) was added. The mixtures were incubated a further 30 min at 37°C and then diluted 5-fold into ice cold DUB buffer. Excess probe was removed by filtration (30K amicon filter) and the combined concentrated lysate (8.4 mg total protein) was stored at -80°C until streptavidin enrichment and MS analysis. A double control also was performed where both the unlabeled and the heavy labeled lysates were incubated with DMSO (1%, vehicle) only prior to addition of Biotin-labeled probe as above. Probe-labeled and control samples were heated at 80°C in 1.2% SDS/PBS for 5 min, diluted to 0.2% SDS/PBS and added to 100 μL of streptavidin-agarose beads (Thermo Scientific). The samples were rotated at room temperature for 3 h, and the beads washed with 0.2% SDS/PBS, 3x PBS and 3x water. The washed beads were resuspended in 6 M urea/PBS, treated with 10 mM dithiothreitol (DTT) (15 min, 65°C) and 20 mM iodoacetamide (room temperature, dark, 30 min), and diluted to 2 M urea/PBS. On-bead trypsin digestion was performed using 2 μg of sequencing-grade trypsin (Promega) overnight at 37°C. LC/LC-MS/MS analysis was performed on an LTQ Orbitrap Discovery mass spectrometer (ThermoFisher) coupled to an Agilent 1200 series HPLC. Digests were pressure loaded onto a 250 μm fused silica desalting column packed with 4 cm of Aqua C18 reverse phase resin (Phenomenex). The peptides were eluted onto a biphasic column (100 μm fused silica with a 5 μm tip, packed with 10 cm C18 and 3 cm Partisphere strong cation exchange resin (SCX, Whatman)) using a gradient 5-100% Buffer B in Buffer A (Buffer A: 95% water, 5% acetonitrile, 0.1% formic acid; Buffer B: 20% water, 80% acetonitrile, 0.1% formic acid). The peptides were eluted from the SCX onto the C18 resin and into the mass spectrometer following the four salt steps outlined in Weerapana et al [89]. The flow rate through the column was set to ~0.25 μL min^-1^ and the spray voltage was set to 2.75 kV. One full MS scan (400-1800 MW) was followed by 8 data dependent scans of the nth most intense ions with dynamic exclusion enabled. The generated tandem MS data from each sample was searched using the SEQUEST algorithm against the human UniProt database. Data sets were searched independently with the following parameter files; for the light search, all amino acids were left at default masses; for the heavy search, static modifications on lysine (+8.0142 m/z) and arginine (+10.0082 m/z) were specified. A static modification of +57 on Cys was specified on all searches to account for iodoacetamide alkylation. The SEQUEST output files generated from the digests were filtered using DTASelect 2.0. Reported peptides were required to be fully tryptic and discriminant analyses were performed to achieve a peptide false-discovery rate below 5%. SILAC-assisted MS data represent two completely independent biological replicates. Quantification of light/heavy ratios “R” was performed using the CIMAGE quantification package as described previously [90]. CIMAGE reports peptide L/H ratios for each unique peptide by both MudPIT run and charge state. These peptides are grouped by protein (Supplementary Table 2) and the median (or average of the two median values for an even number of ratios) peptide L/H ratio is chosen as the representative L/H ratio for that protein. Median values are used instead of averages in order to better filter out outlier peptide ratios. SILAC-assisted MS data represent two completely independent biological replicates (Supplementary Table 1). A number of parameters were used to identify significant PEITC induced changes in protein L/H ratio over control samples. First, the average L/H of two biological runs had to be greater than 4. Second, the L/H ratio had to have a statistically significant p value (p < 0.05) between treated and untreated samples. Third, at least 5 unique tryptic peptides had to have been identified with valid ratios for that protein (Supplementary Table 2).

### Activity profiling

Cell pellets were typically stored at -80 °C until required, at which time they were thawed on ice. Cell lysis was performed in DUB buffer (50 mM K_2_HPO_4_, pH 7.5, 150 mM NaCl, 0.5 mM BME) using a Dounce homogenizer (10 strokes, with grinding, on ice) or by glass beads (as indicated). Lysate (1.5-2 mg/mL) was treated with PEITC (or 1% DMSO control) for 15-25 min, then treated with tagged ubiquitin probe (200 nM – 1.2 μM). Aliquots were quenched in reducing (dithiothreitol) loading buffer. When required, membranes were stripped in 100 mM glycine, pH 4, 500 mM NaCl, 1% SDS, 5 mM BME, at 50 °C for 20 min. For all tagged-ubiquitin probe experiments, samples in loading buffer were warmed to 37 °C prior to electrophoresis.

Activity profiling of HEK293T cells was performed by incubating living cells with PEITC (or 0.1% DMSO control) for 3 h at 37 °C. Cells were harvested and washed two times with ice cold PBS. Cell pellets were then lysed with glass beads (vortexed, 5 × 3 sec bursts at 4 °C) in ice cold lysis buffer (50 mM K_2_HPO_4_, pH 7.5, 150 mM NaCl). The lysate was centrifuged at 17000 g for 10 min at 4 °C and the clarified supernatant (adjusted to 1.5-2 mg/mL) was incubated with Cy5-Ub-VME, TAMRA-Ub-PA or with Biotin-Ahx-Ub-VME/PA (equal mix of both biotin probes, 1.2 μM) for 8 min at 25 °C.

### K63-linked di-ubiquitin gel-based assay

PEITC (1 – 90 μM) was pre-incubated with 150 nM USP1/UAF1 for 8 min at 37°C in assay buffer (50 mM HEPES, pH 7.7, 0.1 mg/mL BSA, 0.5 mM EDTA and 0.5 mM DTT) prior to the addition of K63-linked di-Ub (3 μM). After 10 min at 37°C, the reaction was quenched by the addition of reducing loading buffer, samples resolved on a 15% polyacrylamide gel and stained with InstantBlue (Expedeon).

### Immunoprecipitations

For USP1 immunoprecipitations, HEK293T cells were transiently transfected with an expression plasmid for HA-tagged Ubiquitin (HA-Ubiquitin was a gift from Edward Yeh, Addgene plasmid #18712) using Mirus 2020 according to manufacturer's instructions and confluence at transfection was approximately 60%. 24 h post-transfection, media was aspirated and replaced with fresh media containing either 0.1% DMSO or 0.1% DMSO plus compound and the cells were incubated at 37 °C for 2 h. The cells were harvested, washed in PBS and re-suspended in lysis buffer [40 mM Tris pH 7.4, 140 mM NaCl, 10 mM β-Glycerophosphate, 1 mM EDTA, 0.1% CHAPS, 0.5% NP-40, 50 nM Bortezomib, 1x protease inhibitor cocktail]. After lysis (1x freeze/thaw), protein concentrations were determined (Bradford assay), lysates were adjusted to the same protein concentration (1 mg/mL) and pre-cleared (400 μL lysate) with 10 μL protein G beads (30 min at 4 °C). The beads were removed and the cleared lysate was incubated (with rotation) with anti-USP1 or with normal Rb IgG (negative control) for 2 h at 4 °C. USP1 was immunoprecipitated with protein G magnetic beads (15 μL beads rotated at 1 h at 4 °C. Eluted proteins (5-12 μL, identical volumes of eluent) were analyzed by SDS-PAGE and western blot.

### MCF-7 proliferation assays

In viability assays, Cell Counting Kit-8 (CCK8; Dojindo) was used per manufacture's instruction to measure viability following treatment. MCF-7 cells were seeded in each well of a 96-well plate: 5000 cells/well were seeded for 24 h assays and 3000 cells/well were seeded for 48 h assays. The cells were then cultured for 24 h prior to drug treatment. Cisplatin was dissolved in DMEM immediately before use and PEITC was dissolved in DMSO. Cells were then incubated for either 24 h or 48 h in 100 μL DMEM containing cisplatin, PEITC or a combination of cisplatin and PEITC. 10 μL of CCK-8 solution was added to each well and incubated for 4 h at 37°C. The absorbance of each well at 450 nm was measured using a microplate reader. Relative viability was measured as a percent of untreated control (DMEM only for cisplatin; DMSO [vehicle only] for PEITC and combination treatments). Concentration-response curves were fitted using a log (agonist) versus response – variable slope equation (See Equation 1) using GraphPad Prism which afforded values of EC_50_.

**Equation 1** Y = Abs(minimum) + Abs(maximum-minimum)/(1+10^((LogEC_50_-X)*n))

where X = Log [inhibitor]; Y is cell viability in percent relative to the control and n is the Hill slope.

### Assessment of cisplatin and PEITC synergism

We used the Bliss Model to analyze the effects of PEITC and cisplatin co-treatment [91, 92]. The Combination Index (CI) was calculated according to Equations 2 and 3. Synergism is indicated by a CI of less than 1, additivity by a CI equal to 1, and antagonism by a CI greater than 1.

**Equation 2** CI = E_predicted_/E_Combo_

**Equation 3** E_predicted_ = E_cis_ + E_PE_ – (E_cis_E_PE_)

where E_cis_ is the effect of cisplatin (% inhibition), E_PE_ is the effect of PEITC (% inhibition) and E_Combo_ is the effect of the combined treatment (% inhibition).

Co-treatment also was analyzed using Combenefit software, which assesses the Bliss model and two additional classic drug interaction models, the Loewe and the Highest Single Agent (HSA) models [51].

### His-UCH37 and His-ADRM1 synthesis and purification

Human Adrm1 with an N-terminal 10x histidine tag (addgene plasmid 19423 [59]) was expressed in Rosetta2 BL21(DE3) *Escherichia coli* in high salt (500 mM NaCl) media containing 0.5% glycerol (to limit formation of inclusion bodies). Cultures were induced at an OD_600_ of 0.6 with 0.2 mM IPTG and incubated overnight at 20°C. Human UCH37 (isoform 1) with an N-terminal 6x histidine tag (addgene plasmid 61929 [93]) was expressed in Rosetta BL21(DE3) *Escherichia coli*, induced at an OD_600_ of 0.6 with 0.4 mM IPTG and incubated 2.5 h at 37°C. Cultures were harvested by centrifugation and pellets were resuspended in lysis/wash buffer (20 mM Hepes, Ph 7.6, 200 mM NaCl, 30 mM imidazole [IM], 5 mM TCEP, 5% glycerol), sonicated and lysates clarified by centrifugation. Clarified lysate was incubated with Ni-NTA resin (McLab) overnight at 4°C and His-tagged proteins were eluted by gradient elution with increasing concentrations of IM. Eluted proteins were dialyzed against 20 mM Hepes pH 7.2, 150 mM NaCl, 5 mM TCEP, 1 mM EDTA, 5% glycerol overnight at 4°C and then against storage buffer (20 mM Hepes pH 7.2, 150 mM NaCl, 2 mM DTT, 5% glycerol). Protein concentration determined by Bradford assay. His-ADRM1 was ≥ 98% pure by coomassie stain and western blot and His-UCH37 was ≥ 75% pure by coomassie stain and western blot. Each was used without further purification.

### Reversibility of PEITC-induced inhibition of recombinant UCH37

PEITC (1.5 mM) or DMSO (vehicle only control) was pre-incubated with rUCH37/ADRM1 (1:1.3 molar ratio) (20 nM UCH37) for 15 min at 37 °C in assay buffer (25 mM HEPES, 100 mM NaCl, 0.5 mM EDTA, pH 7.4) prior to the addition of Ub-Rho110MP (150 nM). Just before reaction was initialized, 0 mM or 10 mM DTT was added. Hydrolysis of Ub-Rho110MP was monitored at 37 °C for 15 min by fluorescence (excitation wavelength 492 nm, emission wavelength 525 nm).

### PEITC-UCH37 adduct as determined by mass spectrometry

UCH37 and ADRM1 were mixed in a 1:1.3 molar ratio in assay buffer (25 mM Hepes pH 7.4, 100 mM NaCl, 0.5 mM EDTA. 0.5 mM DTT) and incubated (5 μM UCH37) with 1.5 mM PEITC or with DMSO (vehicle only, control) at 25°C for 2 h at which time samples were frozen and stored at minus 80 °C until MS analysis. For quantitative NEM labeling, 100 μL of 5 μM (~25 μg) UCH37 (plus or minus PEITC) was incubated with 12.5 mM d5-NEM (Cambridge Isotope Laboratories) for 30 min at 25°C. UCH37 samples were then buffer exchanged into PBS buffer using a Micro Bio-Spin 30 chromatography column (Bio-Rad), in order to remove unreacted d5-NEM and PEITC. To each samples 5 μL of 100% trichloroacetic acid was added, the samples were vortexed rapidly and frozen at -80°C for 1 hour. The samples were thawed and the precipitated protein was pelleted by centrifugation at 15k rpm for 10 minutes. The supernatant was removed and the protein pellets were resuspended in 500 μL of ice-cold acetone and pelleted again by centrifugation at 5k rpm for 10 minutes. Pellets were resuspended in 30 μL of 8 M Urea in PBS followed by addition of 70 μL of 100 mM ammonium bicarbonate and 1.5 μL 1M DTT. Samples were incubated at 65°C for 15 minutes. To the samples 2.5 μL of 500 mM d0-NEM (Sigma-Aldrich) (12.5 mM final concentration) was added and the samples were incubated at room temperature. After 30 minutes, 120 μL of PBS was added and the samples were vortexed rapidly. To each sample, 4 μL of 0.5 μg/μL trypsin (Promega), and 2.5 μL 100 mM CaCl_2_ was added and the samples were agitated overnight at 37°C. 10 μL of formic acid was added to each sample, followed by centrifugation at 15k rpm for 20 min to pellet undigested protein. The supernatant was then transferred to a new tube and stored at -20°C until ready for MS analysis.

Samples were analyzed by LC–MS/MS on an LTQ Orbitrap XL mass spectrometer (Thermo Fisher) coupled to an EASY-nLC 1000 nanoLC (Thermo Fisher). 10 μl of each sample were loaded onto 100 μm fused silica column with a 5 μm tip packed with 10 cm of Aqua C18 reverse-phase resin (Phenomenex) using the EASY-nLC 1000 autosampler. Peptides were eluted with a gradient 0–100% buffer B in buffer A (buffer A: 95% water, 5% acetonitrile, 0.1% formic acid; buffer B; 20% water, 80% acetonitrile, 0.1% formic acid). The flow rate through the column was set to 400 nl/min and the spray voltage was set to 3.5 kV. One full MS scan (FTMS) (400–1800 MW) was followed by three high resolution data-dependent scans (FTMS) of the nth most intense ion from an imported mass list of the +3 and +4 ions of the active site cysteine containing tryptic peptide (1316.98, 1318.66, 1320.34, 987.99, 989.25, 990.51) with dynamic exclusion disabled. MS1 and MS2 spectra were manually analyzed.

## SUPPLEMENTARY MATERIALS FIGURES AND TABLES




