# Supplementary material for: Identification of deubiquitinase targets of isothiocyanates using SILAC-assisted quantitative mass spectrometry
Source: Oncotarget. 2017 Apr 20;8(31):51296–316. doi: 10.18632/oncotarget.17261 (PMC5584250; doi:10.18632/oncotarget.17261)
Supplement: Supplementary file 1 [file oncotarget-08-51296-s001.pdf]

Identification of deubiquitinase targets of isothiocyanates using SILAC-assisted quantitative mass spectrometry

SUPPLEMENTARY FIGURES AND TABLES

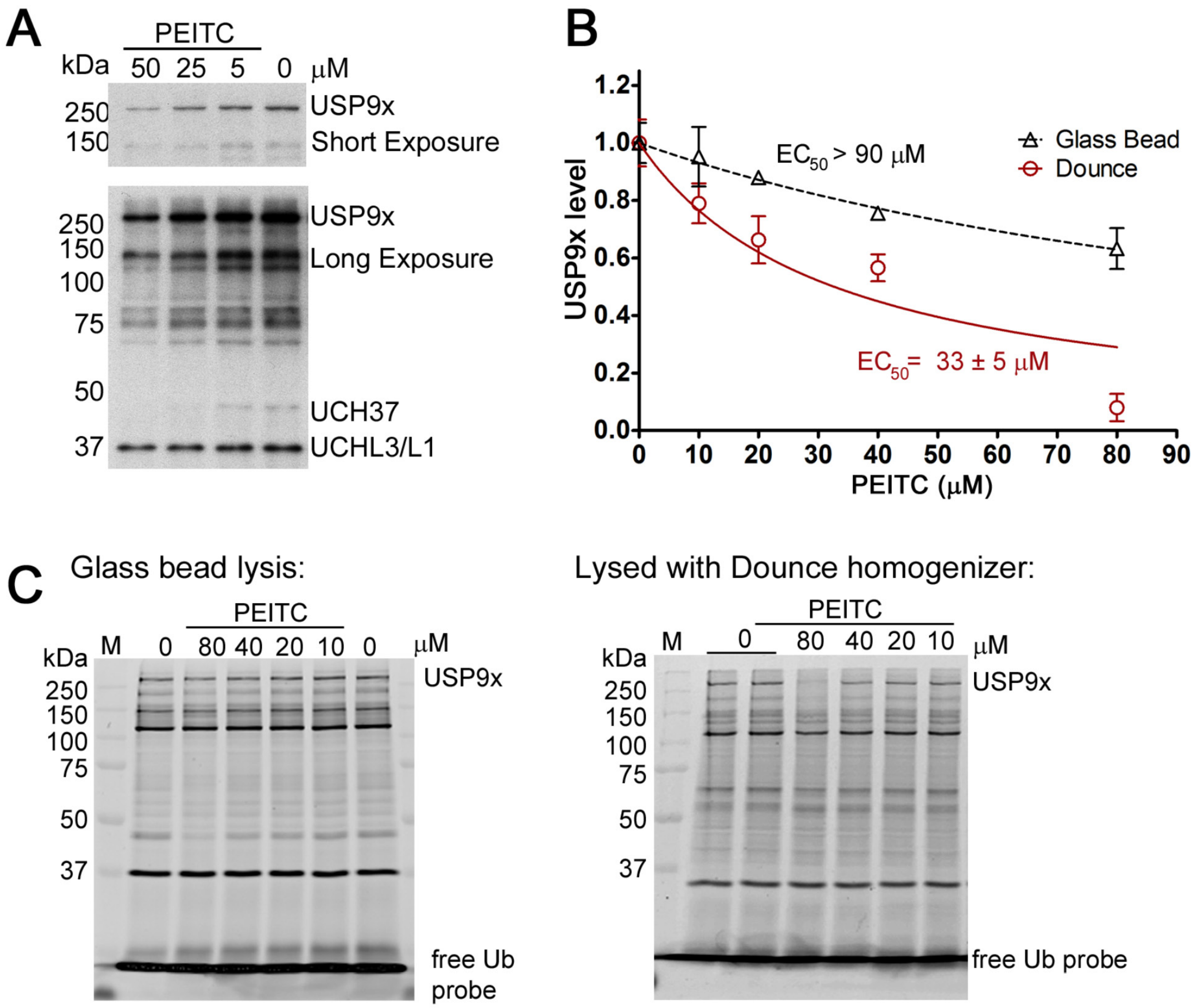

Supplementary Figure 1: USP9x inhibition by PEITC in HeLa cell lysates varies with lysis method and reaction conditions. **(A)** A HeLa cell lysate (dounce homogenization) was treated with PEITC for 15 min followed by a 15 min incubation with HA-Ub-VME. **(B)** HeLa cells were lysed either with glass beads or using a dounce homogenizer. Lysates were adjusted to 1.4 mg/mL and incubated first with PEITC for 25 min, then with Cy5-Ub-VME (400 nM) for 20 min. Lysates were then analyzed by SDS-PAGE and in-gel scan obtained on Typhoon scanner. USP9x levels determined by densitometry. Data presented are the mean ± s.d. of at least two independent experiments. **(C)** Representative scans of experiments described in (B).

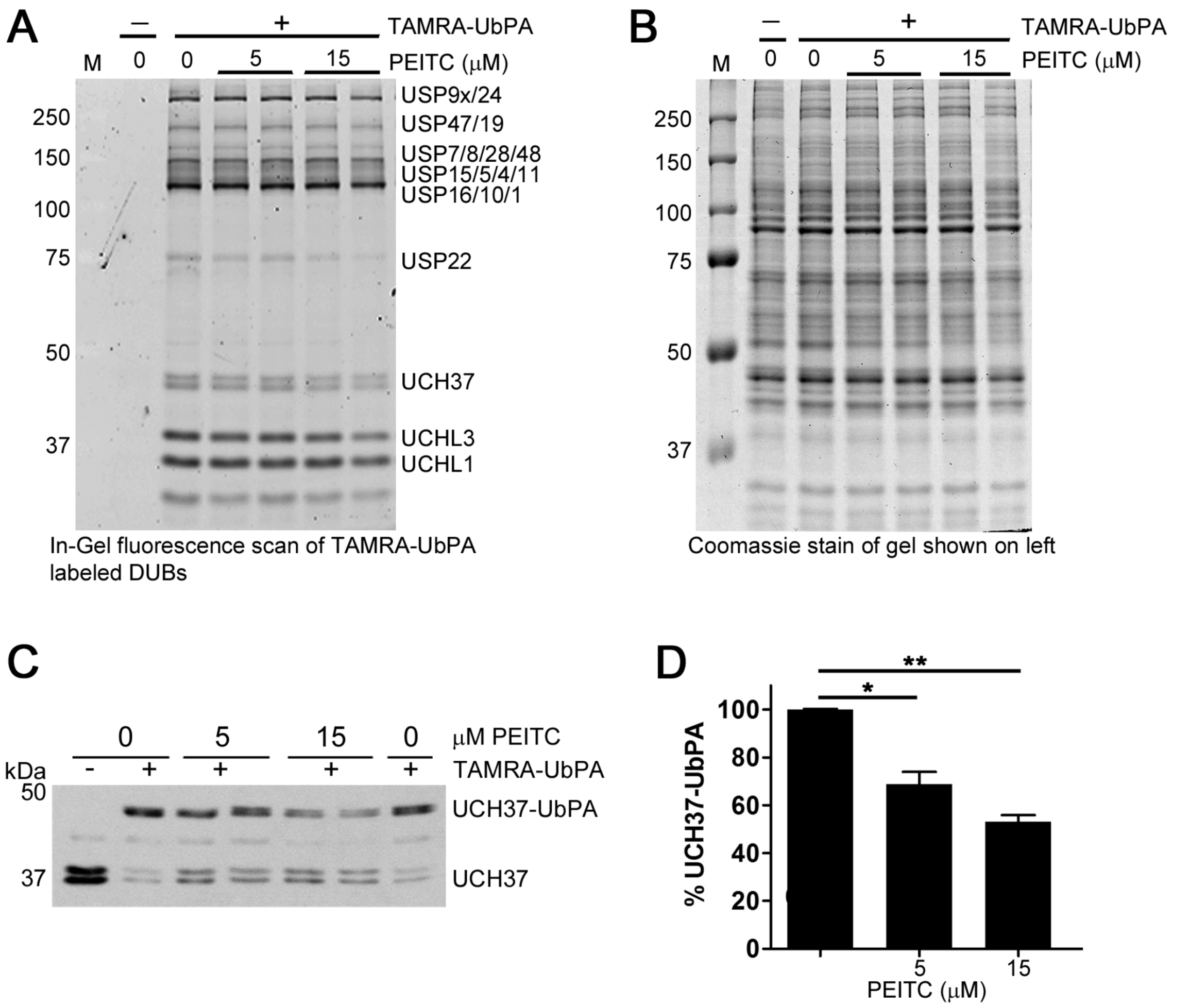

Supplementary Figure 2: PEITC inhibits deubiquitinating enzymes in living cells. **(A-C)** HEK293T cells were incubated with PEITC for 3 h, harvested, washed and lysed with glass beads. Clarified lysates adjusted to 1.6 mg/mL and treated with TAMRA-Ub-PA (1.2 µM) for 8 min at 25°C. In-gel scan depicted in (A) and a Coomassie stain of this gel is shown in (B). DUB assignments shown in (A) based on MW of corresponding DUB. In-gel scan (A) and Coomassie stain (B) both correspond to experiment shown in Figure 3C (top). To obtain the blot shown in (C), the blot depicted in Figure 3C (top) was stripped and reprobed for UCH37. **(D)** Quantification of blot shown in (C). % UCH37-Ub-PA was determined (densitometry) by calculating the ratio of the top, UCH37-probe conjugated band to total UCH37 levels. Data were normalized the DMSO (vehicle only) control. Data represent mean ± SD of 2 independent experiments. $p < 0.05$ *; $p < 0.01$ **; $p < 0.001$ ***.

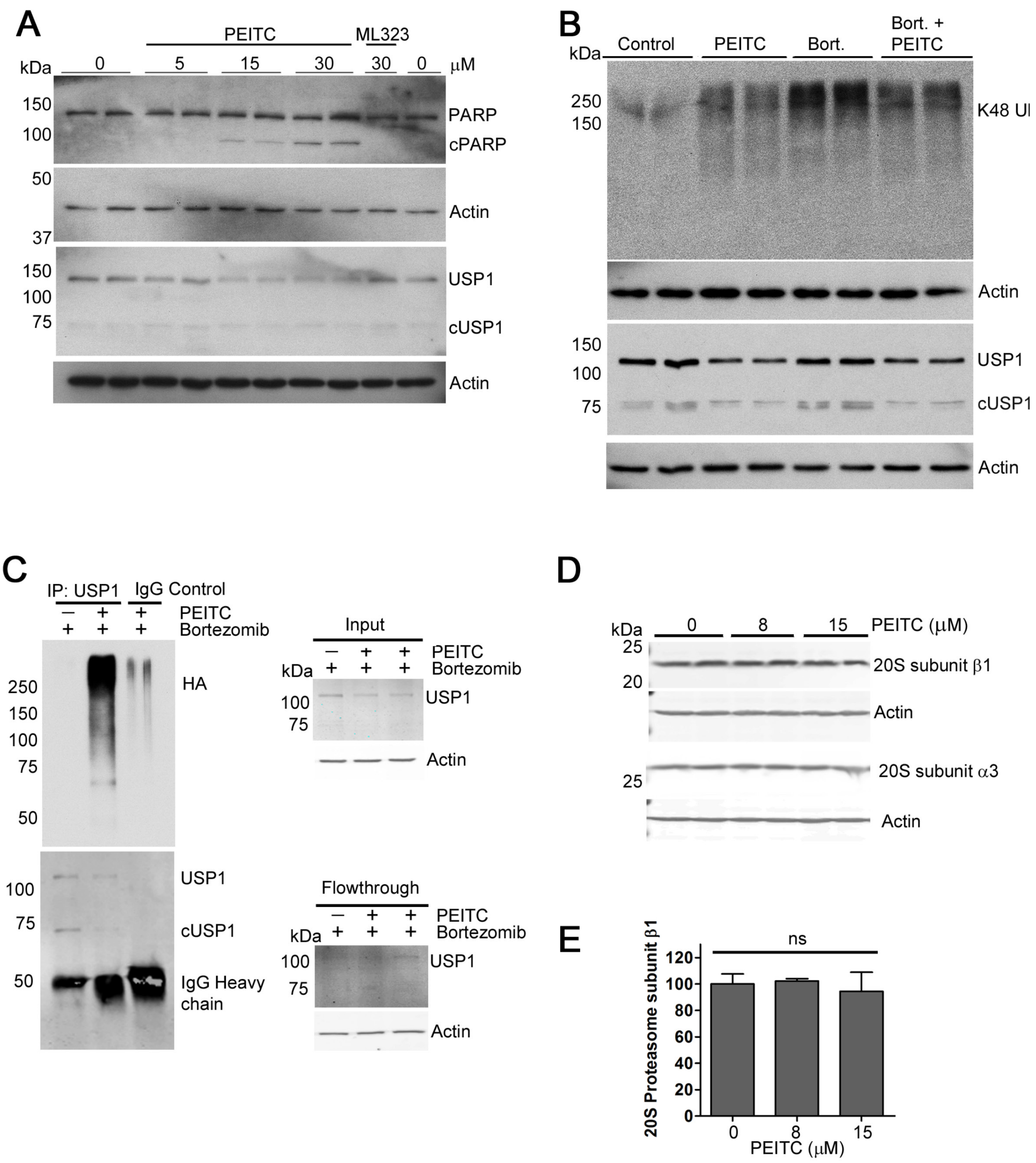

Supplementary Figure 3: PEITC reduces USP1 levels in living cells. (A) HEK293T cells were incubated with PEITC or ML323 for 3 h. Whole cell lysates analyzed by SDS-PAGE and western blot. Data are representative of two independent experiments. **(B)** HEK293T cells were incubated with PEITC (15 µM) and/or bortezomib (bort., 300 nM) for 3 h at 37°C. Whole cell lysates analyzed by SDS-PAGE and western blot. Data represent three independent experiments performed in duplicate. **(C)** HEK293T cells were transiently transfected with HA-Ubiquitin. 24 h post-transfection, cells were incubated with bortezomib (300 nM) or with PEITC (15 µM) together with bortezomib (300 nM) for 3 h at 37°C. USP1 was immunoprecipitated and eluants were probed with anti-HA antibody and anti-USP1 antibody. **(D)** HEK293T cells were treated with PEITC for 3 h at 37°C . WCLs were analyzed by SDS-PAGE and western blot. Actin is shown as a loading control. **(E)** Quantification of blot shown in (D). 'ns' = not significant.

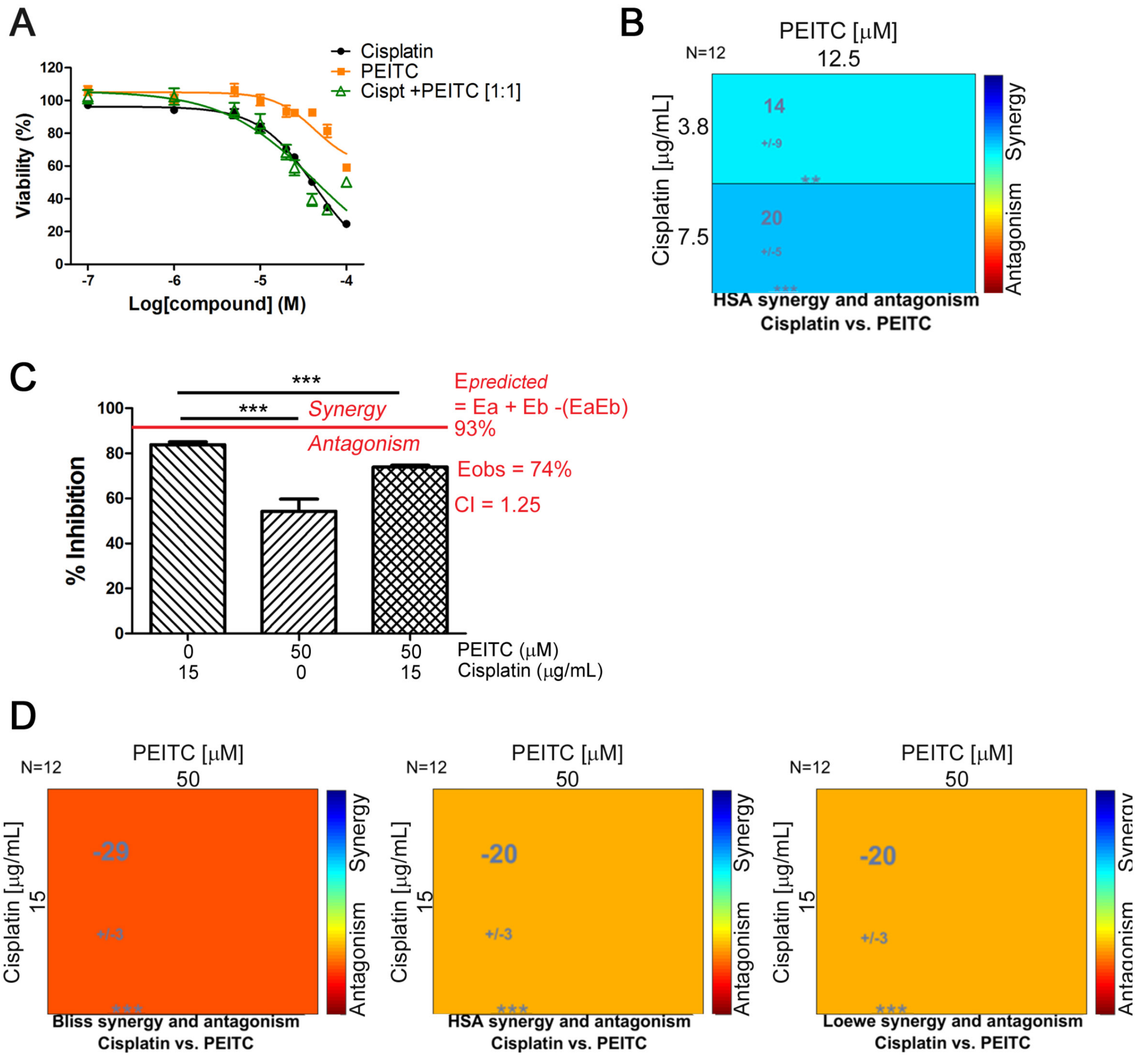

Supplementary Figure 4: PEITC treatment is synergistic with cisplatin at low concentrations but antagonistic at higher concentrations. **(A)** MCF-7 cells were incubated with PEITC, cisplatin or with a 1:1 combination of PEITC and cisplatin for 24 h. Cell viability was measured using the CCK-8 assay. Data represent at least 4 replicates ($n \geq 4$). **(B)** MCF-7 cells were treated with 12.5 µM PEITC in combination with 3.8 or 7.5 µg/mL cisplatin for 48h. Inhibition of cell growth/viability was measured using the CCK-8 assay and drug interaction was analyzed using Combenefit software. Data represent quadruplicate samples from 3 independent experiments ($n = 12$). **(C)** MCF-7 cells were treated with 50 µM PEITC, 15 µg/mL cisplatin or with a combination of 50 µM PEITC and 15 µg/mL cisplatin for 48h. Inhibition of cell growth/viability was measured using the CCK-8 assay and CI values were determined using the Bliss independence model as described in the materials and methods. Data represent quadruplicate samples from 3 independent experiments ($n = 12$). **(D)** As in (C), drug interaction was analyzed using Combenefit. Data represent quadruplicate samples from 3 independent experiments ($n = 12$). 'ns' equals not significant. $P < 0.05$ *; $P < 0.01$ **; $P < 0.001$ ***.

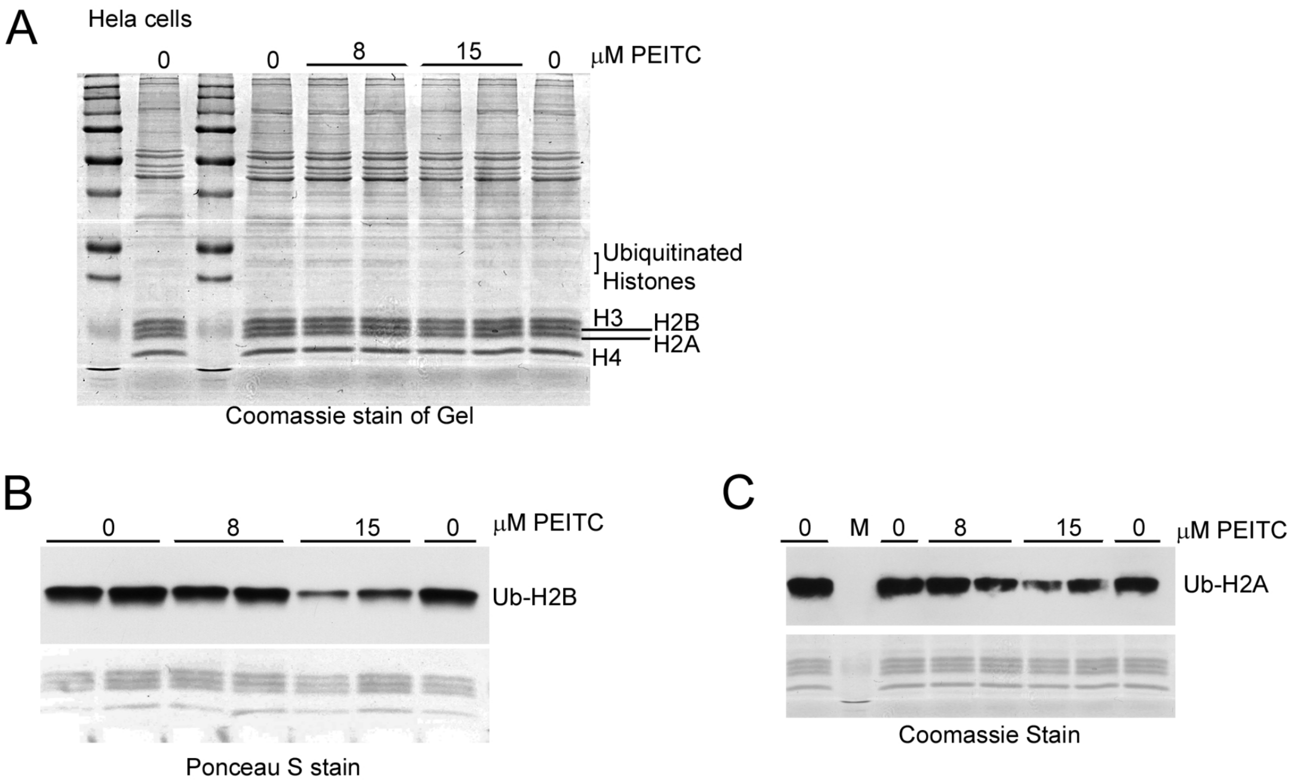

Supplementary Figure 5: PEITC decreases Ub-H2A and Ub-H2B levels in HeLa cells. **(A-C)** HeLa cells were incubated with PEITC or with DMSO (vehicle only) for 3h. Cells were harvested and subjected to standard histone extraction protocol, resolved on a 14% polyacrylamide gel and stained with InstantBlue (Expedeon, (A). (B) and (C) Histone extracts (equal protein load for each lane) were analyzed by SDS-PAGE and immunoblotted with anti-Ub-H2A and anti-Ub-H2B. Ponceau stain (B) or Coomassie (C) are shown to demonstrate loading. Data are representative of two independent experiments

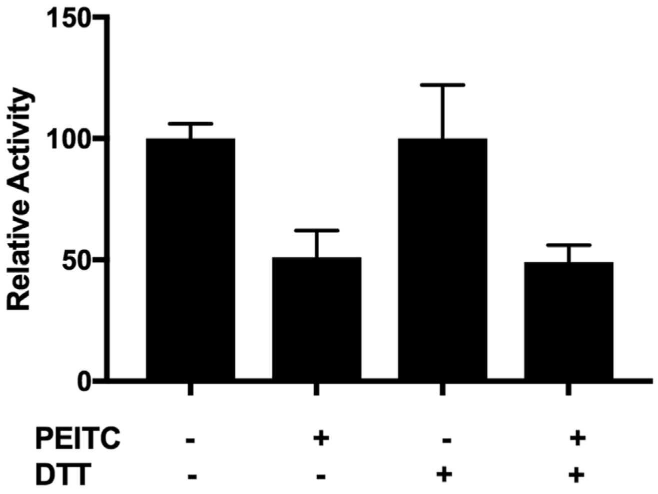

Supplementary Figure 6: Inhibition of rUCH37/ADRM1 by PEITC in the presence and absence of dithiothreitol. PEITC (1.5 mM) or DMSO (vehicle only control) was pre-incubated with rUCH37/ADRM1 (20 nM) for 15 min at 37 °C in assay buffer (25 mM HEPES, 100 mM NaCl, 0.5 mM EDTA, pH 7.4) prior to the addition of Ub-Rho110MP (150 nM). Just before reaction was initialized, 0 mM or 10 mM DTT was added. Hydrolysis of Ub-Rho110MP was monitored at 37 °C for 15 min by fluorescence (excitation wavelength 492 nm, emission wavelength 525 nm).

Supplementary Table 1: Activity profiling of DUB inhibition by PEITC. SILAC-assisted MS data was collected from two biological replicates for both control (-PEITC) and treated (+PEITC) samples. Only those DUBs that had L/H SILAC ratios in both biological replicates (n=2) are reported. DUBs only identified with light signals in the PEITC treated samples are assigned an arbitrary value of 20. Those DUBs with statistically significant ($p < 0.05$) increases in their average L/H ratio in PEITC treated over control samples are highlighted in red.

See Supplementary File 1

Supplementary Table 2: Representative chromatograms for DUBs identified from +/-PEITC treated SILAC-assisted MS analysis. For each of the 35 DUBs, extracted ion chromatograms and isotopic envelopes of a representative target peptide m/z with both light (-PEITC) and heavy (+PEITC) modifications are colored in red and blue, respectively. Additionally, for each protein, a list of all unique peptides and their corresponding L/H ratios identified from a single mass spectrometry run is shown.

OTUB1

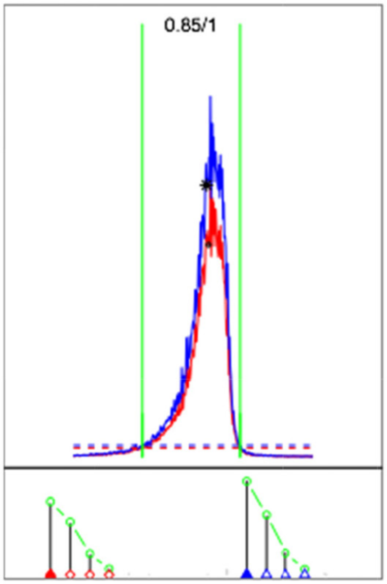

Peptide	L/H Ratio
F.GFSHLEALLDDSK.E	0.85
F.TIEDFHNTFMDLIEQVEK.Q	0.7
K.FFEHFIEGGR.T	0.85
K.QTSVADLLASFNDQSTSDYLVVYLR.L	0.81
R.AFGFSHLEALLDDSK.E	0.86
R.AFGFSHLEALLDDSKELQR.F	0.84
R.GEGGTTNPHIFPEGSEPK.V	0.97
R.IQQEIAVQNPLVSER.L	0.97
R.LELSVLYK.E	0.86
R.LLTSGYLQR.E	0.83

USP25

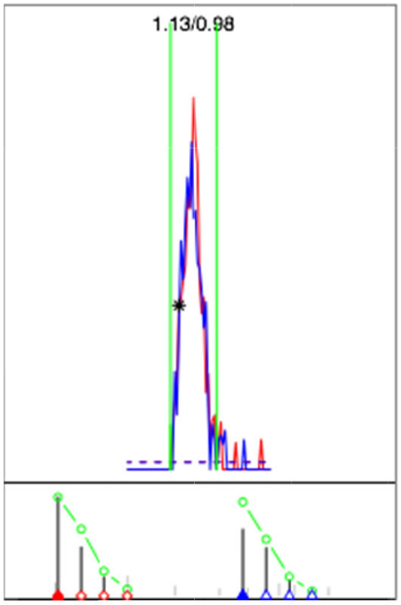

Peptide	L/H Ratio
E.LPPVLTFELSR.F	1.62
K.HLKEETIQIITK.A	1.13
K.HQQTFLNQLR.E	1.15
K.LEFPQVLYLDR.Y	1.21
K.LLDWLEDAFQMK.A	1.12
K.SGQEHWFTELPPVLTFELSR.F	1.16
R.AIALSLAESNR.A	1.82
R.ESYIDSLLFLICAYQNNK.E	1.19
R.FLAVGVLEGK.K	1.13
R.YISVGSQADTNVIDLTGDDKDDLQR.A	1.14
R.YLFALLVGTK.R	1.13

(*Continued*)

USP8

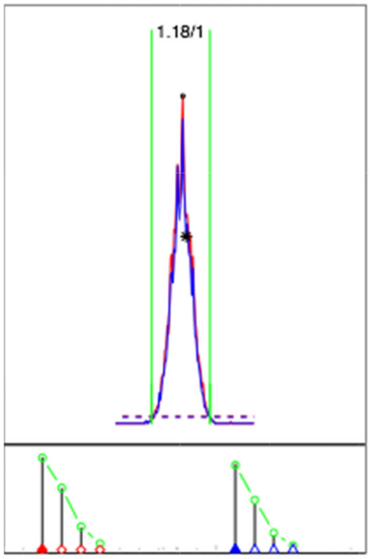

Peptide	L/H Ratio
K.AKPQIPAER.D	1.32
K.ALWTGQYR.Y	1.09
K.FDDHEVSDISVSSVK.S	1.14
K.FLDPITGTFR.Y	1.18
K.GEVAEEFGIIMK.A	1.06
K.INDQFAGYSQQDSQELLLFLMDGLHEDLNK.A	1.36
K.LQTSVDFPLENLDLSQYVIGPK.N	1.42
K.NISLIIMDAR.R	1.13
K.QLNESIIVALFQGQFK.S	1.17
K.QQQDYFHSILGPGNIK.K	1.27
K.STVQCLTCHKK.S	1.12
K.YNLFSVSNHYGGLDGGHYTAYCK.N	1.17
R.GNVEYVVLLDWFSSAK.D	1.35
R.IHAETALLMEK.N	1.34
R.KPTVTPTVNR.E	1.25
R.MGPLNISTPVEPVAASK.S	1.23
R.NEPLVLEGGYENWLLCYPQYTTNAK.V	1.48
R.NLNPVFGGSGPALTGLR.N	1.27
R.STKPVVFSPTLMLTDEEK.A	1.24
R.SYSSPDITQAIQEEEKR.K	1.77
R.TFEAFMYLSLPLASTSK.C	1.21

USP13

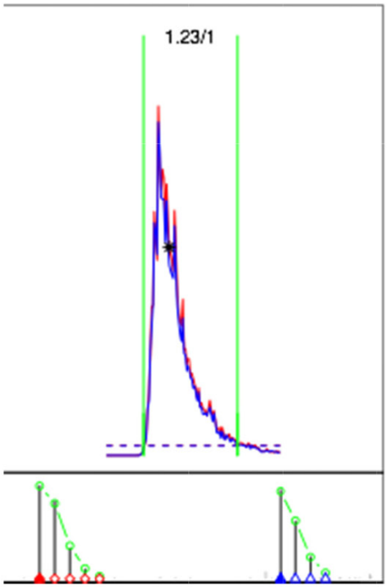

Peptide	L/H Ratio
K.FTFGLDWVPK.K	1.18
K.KFTFGLDWVPK.K	1.23

(*Continued*)

USP5

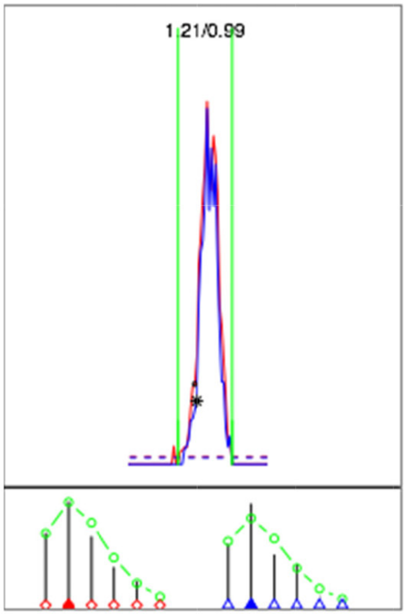

Peptide	L/H Ratio
F.FLHLINMVER.N	1.15
F.SSCLEAYGAPEQVDDFWSTALQAK.S	1.21
H.MGTSTMCGHYVCHIK.K	1.22
K.DLGYIYFYQR.V	1.21
K.FTFGLDWVPK.K	1.23
K.GHPEFSTNR.Q	1.28
K.IFQNAPTDPTQDFSTQVAK.L	1.66
K.IVILPDYLEIAR.D	1.39
K.KFTFGLDWVPK.K	1.23
K.KLDVSIEMPEELDISQLR.G	1.23
K.LDVSIEMPEELDISQLR.G	1.47
K.LGHGLLSGEYSKPVPESGDGER.V	1.37
K.LGHGLLSGEYSKPVPESGDGERVPEQK.E	1.42
K.LGTITPDGADVYSYDEDDMVLDPSLAEHLSHFGI	1.27
K.MALPELVR.A	1.28
K.TMTELEIDMNQR.I	1.23
K.VCASEKPPK.D	1.41
K.VCASEKPPKDLGYIYFYQR.V	1.39
K.YQLFAFISH.M	1
K.YQLFAFISHMGTSTM.C	1
K.YQLFAFISHMGTSTMCGHY.V	1
R.AQVPFSSCLEAYGAPEQVDDFWSTALQAK.S	1.34
R.AVDWIFSHIDDLDAEAAMDISEGR.S	1.88
R.DRVTSAVEALLSADSASR.K	1.24
R.ENLWLNLTDGSILCGR.R	1.21
R.ETGYPLAVK.L	1.84
R.FASFPDYLVIQIK.K	1.51
R.GTGLQPGEEELPDIAPPLVTPDEPK.G	2.01
R.IGEWELIQESGVPLKPLFGPGYTGIR.N	1.36
R.IPPCGWK.C	1.33
R.KQEVQAWDGEVR.Q	1.24
R.KYVDKLEK.I	1.28
R.LAIGVEGGFDLSEEK.F	0.99
R.LAIGVEGGFDLSEEKFELDEDVK.I	1.32
R.QQDAQEFFLHLINMVER.N	1.19
R.SAADSISESVPVGPK.V	1.38
R.SSENPNEVFR.F	1.35
R.VDYIMQLPVPMDAALNK.E	1.29
R.VDYIMQLPVPMDAALNKEELLEYEEK.K	1.3
R.VTSAVEALLSADSASR.K	1.34
R.WVIYNDQK.V	1.13
R.YFDGSGGNNHAVEHYR.E	1.22
T.SPMLDESVIIQLVEMGFPMDACR.K	1.25
T.SPMLDESVIIQLVEMGFPMDACRK.A	1.09
Y.ICMNTFLGFGK.Q	1.22

(*Continued*)

USP7

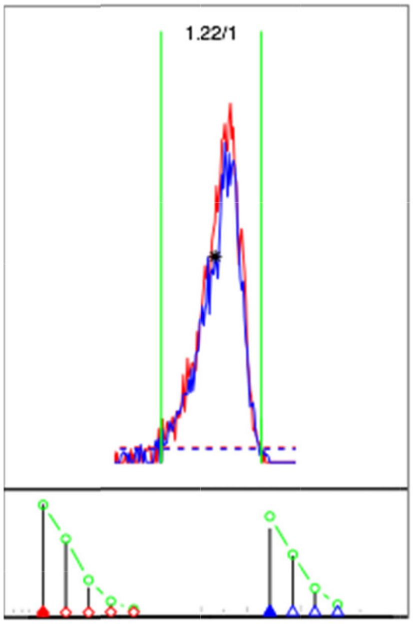

Peptide	L/H Ratio
K.ALDELMDGDIIVFQK.D	1.24
K.ALDELMDGDIIVFQKDDPENDNSELPTAK.E	1.42
K.CIWLNSQFR.E	1.22
K.ENDWGFSNFMAWSEVTDPEK.G	1.42
K.FAIVMMGR.H	1.22
K.FDKDHDVMLFLK.M	1.24
K.IIGVHQEDELLECLSPATSR.T	1.28
K.INDRFEFPEQLPLDEFLQK.T	1.32
K.IRDLLPVMCDR.A	1.3
K.KLYYQQLK.M	1.3
K.NIFESFVDYVAVEQLDGDNK.Y	1.27
K.NIFESFVDYVAVEQLDGDNKYDAGEHGLQEAEK.	1.24
K.NSSLAEFVQSLSQTMGFPQDQIR.L	1.21
K.NSSLAEFVQSLSQTMGFPQDQIR.L	1.28
K.SFGWETLDSFMQHDVQELCR.V	1.2
K.SVGFFLQCNAESDSTSWSCHAQAVLK.I	20
K.TMIELSDNENPWTIFLETVDPELAASGATLPK.F	1.45
K.WCKFDDDVVSR.C	1.02
N.SLLQTLFFTNQLR.K	1.11
R.AGFIQDTSLILYEEVKPNLTER.I	1.27
R.DLLQFFKPR.Q	1.37
R.FYPDRPHQK.S	1.4
R.HQYINEDEYEVNLK.D	1.3
R.IHQGEHFR.E	1.23
R.IQSLLDIQEK.E	1.26
R.IQSLLDIQEKEFEK.F	1.33
R.ITQNPVINGNVALSDGHNTAEEDMEDDTSWR.S	1.31
R.KAVYMMPTEGDDSSK.S	1.27
R.LLEIVSYK.I	0.8
R.LLEIVSYK.I	1.17
R.LNTDPMLLQFFK.S	0.91
R.LSESVLSPPCFVR.N	1.27
R.MNYFQVAK.T	1.11
R.PWLGLDHFNK.A	1.32
R.SLNYCGHIYTPISCK.I	1.36
R.VFYELQHSDKPVGTK.K	1.28
R.VFYELQHSDKPVGTKK.L	1.32

(*Continued*)

USP9Y

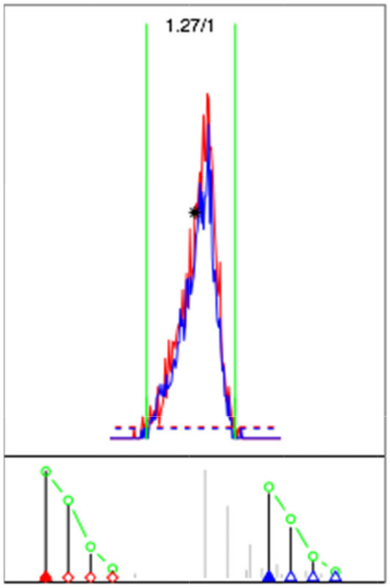

Peptide	L/H Ratio
K.ALTLQDLDNIWAAQAGK.H	1.2
K.CLAENAVYLCDR.E	1.31
K.ELLAEQTSEKK.Y	1.22
K.FGTLNGFQILHDR.F	1.16
K.FNDYFEFPR.E	1.27
K.GIPDDRDGLFDTIQR.S	1.35
K.LIGQLNLK.D	1.31
K.LLLTAIGYGHVR.A	1.15
K.LMGDEPDLDPDINKDFFESNVLQLDPSLLTENG	1.38
K.LYSVVSQLIR.C	1.23
K.MDDDEEMKNQCFGGEYMGEVFDHMMK.R	1.25
K.RFDYDWER.E	1.47
K.VISSVSYYTHR.H	1.17
K.WTWAVEWLGDELER.R	1.28
R.FLFTTGFHTK.K	1.25
R.KLIGQLNLK.D	1.35
R.LLQISSENGK.M	1.3
R.LVGVLVHSGQASGGHYY.S	1
R.LVGVLVHSGQASGGHYYSY.I	1
R.RGAYLNALK.I	1.36
R.TNDKWVIPALK.Q	1.27
R.WVVPVLPK.G	1.32

(*Continued*)

USP9X

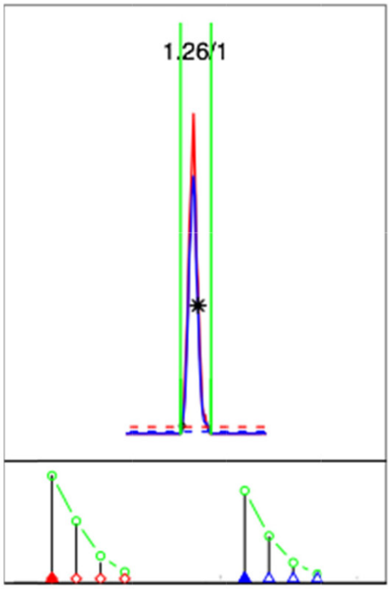

Peptide	L/H Ratio
K.ALGHPAMLSK.V	1.26
K.ALTLQDLDNIWAAQAGK.H	1.2
K.CLAENAVYLCDR.E	1.31
K.ELIDDFIFPASNVYLQYMR.N	1.35
K.ELLAFQTSEKK.F	1.22
K.FGTLNGFQILHDR.F	1.16
K.FNDYFEFPR.E	1.27
K.GELEVLLEAAIDLSK.K	1.31
K.GIPDDRDGLFDTIQR.S	1.35
K.HLSFVVR.F	1.23
K.HSGDYFTLLR.H	1.25
K.LAWDFSPEQLDHLFDCFK.A	1.22
K.LIGQLNLK.D	1.31
K.LLLTAIGYGHVR.A	1.15
K.LMGDEPDLDPDINKDFFESNVLQLDPSLLTENG	1.38
K.LPPVLAIQLK.R	1.53
K.LSVPATFMLVSLDEGPGPPIK.Y	1.56
K.LYSVVSQLIR.C	1.23
K.MDDDEEMKNQCFGGEYMGEVFDHMMK.R	1.25
K.RFDYDWER.E	1.47
K.SGGLPLVLSMLTR.N	1.3
K.VISSVSYYTHR.H	1.17
K.WTWAVEWLGDELER.R	1.28
R.AVAEACQPGVEGVNPMTQINQVTHDQAVVLQSA	1.5
R.AYMMDDLELIGLDYLWR.V	1.2
R.FLFTTGFHTK.K	1.25
R.GSASDWYDALCILLR.H	1.26
R.HGNPEEEEWLTAER.M	1.33
R.IKANVAHTK.I	1.2
R.KLIGQLNLK.D	1.35
R.LLQISSFNGK.M	1.3
R.LVGVLVHSGQASGGHYY.S	1
R.LVGVLVHSGQASGGHYYSY.I	1
R.LWGEPVNLR.E	1.6
R.MAEWIQQNNILSIVLR.D	1.19
R.MQYSMEYFQFMK.K	1.23
R.NHQNLLDSLEQYVK.G	1.09
R.QVAQEQFFLMCTR.C	1.29
R.RGAYLNALK.I	1.38
R.RPYTGNPQYTYNNWSPPVQSNETSNGYFLER.S	1.43
R.TGETGIEETILEGHLGVTK.E	1.35
R.TNDKWVIPALK.Q	1.27
R.WVVPVLPK.G	1.32
R.YISFLWQVADLGSSLNMPPLR.D	1.32

(*Continued*)

USP15

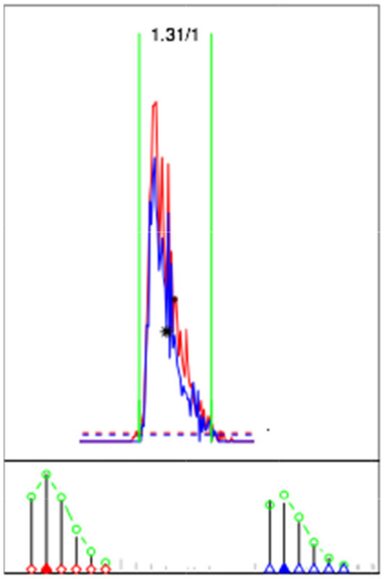

Peptide	L/H Ratio
K.DGDAQSLKEHLIDELDYILLPTEGWNK.L	1.57
K.EHLIDELDYILLPTEGWNK.L	1.23
K.IFSIPDEK.E	1.46
K.IFSIPDEKETR.L	1.19
K.IGNILDLCTALSALSGIPADK.M	1.42
K.ISVTFDPFCYLTLPLPMK.K	1.34
K.ISVTFDPFCYLTLPLPMKK.E	1.31
K.LDLWSLPPVLVVHLK.R	1.35
K.LKDCIELFTTK.E	1.25
K.LVSWYTLMEGQEPIAR.K	1.39
K.LYNLLLLR.M	1.2
K.MIVTDIYNHR.F	1.18
K.VVAEEAWENHLK.R	1.24
K.VVAEEAWENHLKR.N	1.28
K.YMSNTFEPLNKPDSTIQDAGLYQGQVLVIEQK.N	1.36
K.YQEELNFDNPLGMR.G	1.3
K.YVGFDSWDKYQMGDQNVYPGPIDNSGLLK.D	1.3
N.ILDLCTALSALSGIPADK.M	1.61
R.DDIYVFEININR.T	1.35
R.DKLDTLVDFPINDLDMSEFLINPNAGPCR.Y	1.42
R.FAPQFSGYQQQDCQELLAFLLDGLHEDLNR.I	1.26
R.IFAMDENLSSIMER.D	1.44
R.KGDTWYLVDSR.W	1.35
R.KIFSIPDEK.E	1.28
R.LFTFQFNNLGNTDINYIK.D	1.36
R.LFTFQFNNLGNTDINYIKDDTR.H	1.25
R.MDPLTKPMQYK.V	1.25
R.NDSIIVDIFHGLFK.S	1.28
R.NDSIIVDIFHGLFK.S	1.21
R.NNTEDKLYNLLLLR.M	1.38
R.SFLALDWDPDLK.K	1.34
R.TEDTEHVIIPVCLR.E	1.24
R.TLEVYLVR.M	1.31
R.YNLIAVSNHYGGMGGGHYTAF.A	1

(*Continued*)

USP47

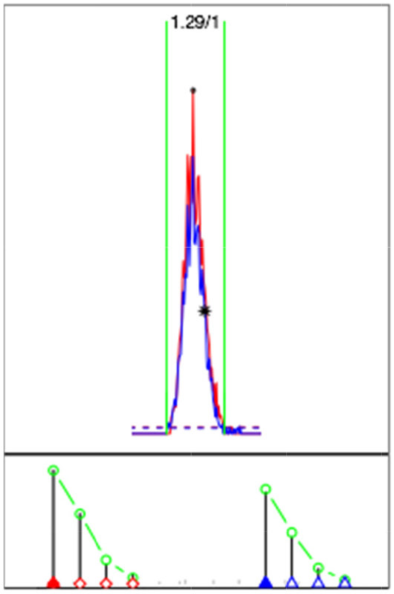

Peptide	L/H Ratio
I.RLFVLLPEQSPVSYSK.R	1.33
K.FLEVDEYPEHIK.N	1.29
K.FLLDAVFAK.G	1.29
K.LDPFQEVVLESSSVDELR.E	1.39
K.LDPFQEVVLESSSVDELREK.L	1.49
K.LFCLHPTK.Q	1.27
K.LSEISGIPLDDIEFAK.G	1.35
K.MMDLEEVIPLDCCR.L	1.3
K.NFLHLTDK.D	1.24
K.NFLHLTDKDGEQPQILLEDSSAGEDSVHDR.F	1.23
K.NSLIYELFSVMVH.S	1
K.RFDFDYTTMHR.I	1.27
K.SFSDEQWYSFNDQHVSR.I	1.25
K.SLLDANFEPGKK.N	1.28
K.SMSQLAVLSR.R	1.22
K.STYMFDLLLETR.K	1.28
K.SVEAILEESTEK.L	0.94
K.VHVVDLK.A	1.17
K.VSTLNVWPLYICDDGAVIFYR.D	1.32
K.WEFEESEEDPVTSIPYQLQR.L	1.42
K.WKQTEQADLINELYQGK.L	2.47
K.WLMVHVDK.R	1.28
K.YDEFHDYLER.S	1.28
R.DKTEELMELTDEQR.N	1.42
R.FDFDYTTMHR.I	1.27
R.FIGPLPR.E	1.38
R.GTFPCDISVLDIHQDLDWNPK.V	1.32
R.ITLNLPASTPVR.K	1.36
R.KLFEDVANK.V	1.25
R.KPDQVFQSYKPGEVMVK.V	1.37
R.LFVLLPEQSPVSYSK.R	1.38
R.LFVLLPEQSPVSYSKR.T	1.33
R.LFVLLQTSK.K	1.27
R.SFGWDSSEAWQQHDVQELCR.V	1.19
R.SYEGEEDTPMGLLLGGVK.S	1.44
R.VKGPVGSLK.S	1.27

(*Continued*)

USP24

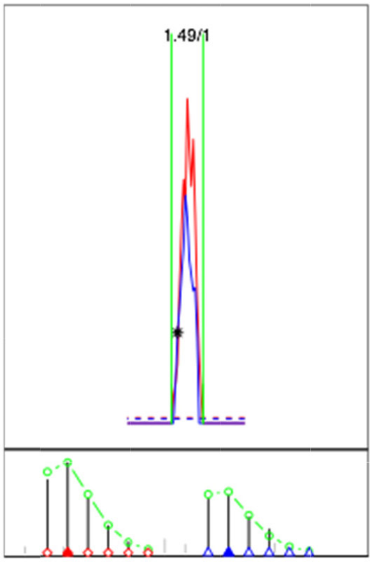

Peptide	L/H Ratio
K.AGGLSLVVNVMQR.D	1.54
K.CLLASTYLAR.L	1.19
K.DSLIAGEALSLLVTCLQLR.S	1.4
K.EMLGSSLIKPLLDDFLFR.A	1.55
K.FGELGGFAAIQAK.L	1.27
K.FLVTLAQK.C	1.28
K.IAMESPDEEIANEAIQLIINYSYINLNPR.L	1.39
K.LQYYVPENFWK.I	1.66
K.LVTGSLIACHR.L	1.39
K.QQHQPSASSILESLFR.S	1.33
K.SSQLNNPQFVWVVPALR.Q	1.49
K.TFFENVNLCDHR.L	1.34
R.EQQDAYEFFTSLIDQMDEYLKK.M	1.45
R.FGFDWESGR.S	1.3
R.FLLVGQTMPTLLDEDLTK.D	1.49
R.FPWMLNMEPYTVSGMAR.Q	1.29
R.IILNSHSPAGSAAISQQDFHPK.C	1.39
R.LEAASSALGGPTLTHAVTR.A	1.52
R.LLSQCMEYFDLR.C	1.17
R.LVSIPELLSAVK.L	1.48
R.NWAEVFGEGNMFAVSPVSTFQK.E	1.52
R.TAECETSEADNILLAGHLR.L	1.43
R.VLTDHWSIPYKR.E	1.35
R.YVITIEDFYSVPR.T	1.67

UCHL3

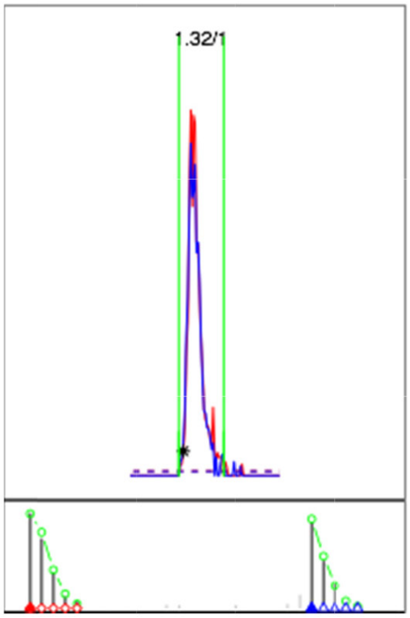

Peptide	L/H Ratio
K.DKMHFESGSTLKK.F	1.32
K.FLEESVSMSPEER.A	1.35
K.KFLEESVSMSPEER.A	1.48
K.KFMERDPDELR.F	1.31
K.MHFESGSTLK.K	1.34
K.QLGLHPNWQFVDVY.G	1
K.QLGLHPNWQFVDVYGMDPELLSMVPR.P	1.54
K.QTISNACGTIGLIHAIANNK.D	1.32
K.QTISNACGTIGLIHAIANNKDK.M	1.23
K.SQGQDVTSSVYFMK.Q	1.26
K.YEVFRTEEEEK.I	1.26
R.KPFPINHGETSDETLLEDAIEVCK.K	1.49
R.PVCAVLLLFPITEK.Y	1.44
R.PVCAVLLLFPITEKY.E	1.41
R.VTHETSAHEGQTEAPSIDEK.V	1.46
R.WLPLEANPEVTNQFLK.Q	1.39
R.YLENYDAIR.V	1.35
Y.GMDPELLSMVPR.P	1.47

(*Continued*)

USP28

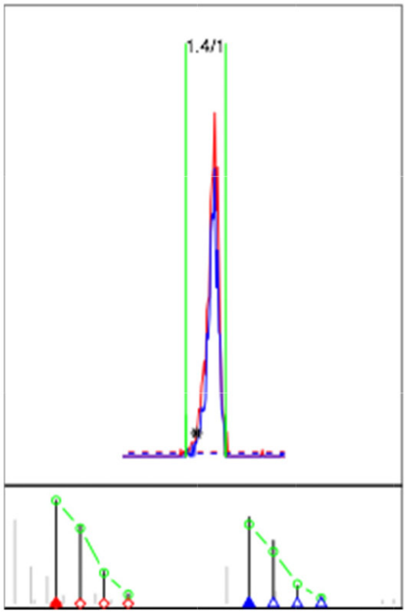

Peptide	L/H Ratio
K.FVDPSAALDLLK.G	1.4
K.LEFPQIIYMDR.Y	1.36
K.LPPVLTFELSR.F	1.62
R.FAAVMESIQGVSTVTVK.-	1.3
R.FPLPDMLK.Y	1.43

USP19

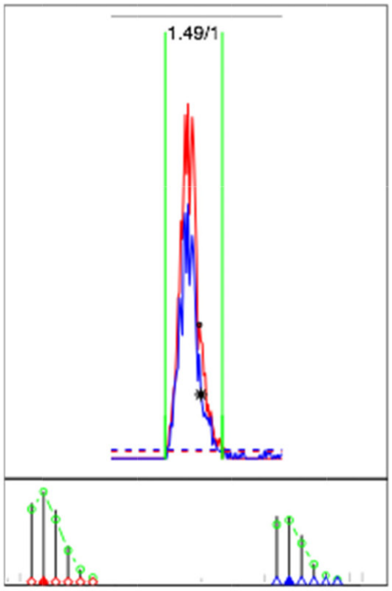

Peptide	L/H Ratio
K.EGACEDPHDLLATPTPELLLDWR.Q	1.78
K.ENSTASEVLDSLSQSVHVKPENLR.L	1.28
K.GLCRPENIGYPFLVSVPASR.L	1.49
K.GSLLHLTLPK.K	1.45
K.VAVPTGPTPLDSTPPGGAPHPLTGQEEAR.A	1.75
K.VLPVFYFAR.E	1.35
K.VSITFDPFLYLPVPLPQK.Q	1.48
R.DFFHDR.S	1.38
R.DKINDLVEFPVR.N	1.45
R.EQDFTLIFQTR.D	1.59
R.IQNKPYTETVDSDGRPDEVVAEEAWQR.H	1.47
R.LAQLLEGYAR.Y	1.28
R.LEDKGDTPLELGDDCSLALVWR.N	1.31
R.LPNVLIVQLK.R	1.53
R.NDSFIVDLFQGQYK.S	1.29
R.VFLPSHSLDTVSPSDTLLCFELLSSELAK.E	1.41
R.VGVGPLQLEDVDAAFTDTDCVVR.F	1.38
R.VVVLEVQQRPQVPSVPISK.C	1.76
R.VWAAPDRGPVPSTSGISSEMLASGPIEVGSLPA	1.51
R.YAYVLFYR.R	1.3
R.YSVSVFQPPFQPGR.M	1.89

(*Continued*)

OTUD7B

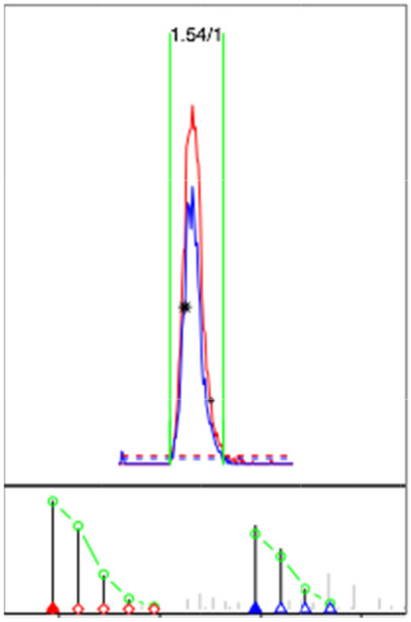

Peptide	L/H Ratio
K.FIFVGTLK.M	1.33
K.LLPLHFAVDPGK.G	1.54
K.NMGGLMHSK.G	1.38
K.NWDVNAALSDFEQLR.Q	1.37
K.QPNCSFYGHPETNNFCSCCYR.E	1.68
R.DLIEQSMLVALEQAGR.L	1.5
R.DSGGEAFAPIPFGGIYLPLEVPASQCHR.S	1.59
R.GALLPPPYR.V	1.74
R.GISHASSSIVSLAR.S	1.39
R.LASVILSLEVK.L	1.45
R.MHLGTNGANCGGVESSEEPVYESLEEFHVF.V	1
R.QLAGGPCVGGLPPYATFPR.Q	1.78
R.RPIVVVADTMLR.D	1.56
R.SPLVLAYDQAHFSALVSMEQK.E	2.31

USP38

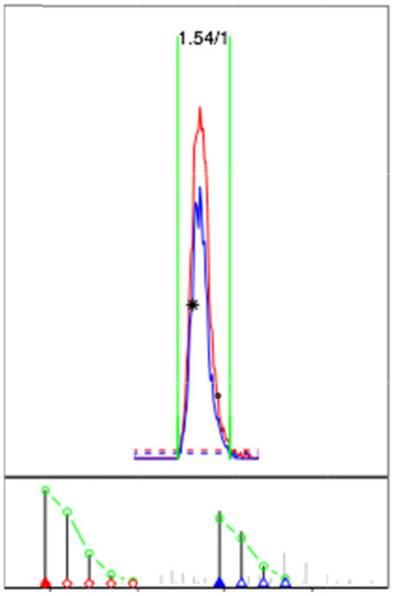

Peptide	L/H Ratio
R.LSDLLTDFVQCIPK.G	1.56
K.ILDNVSLPLVLELPVKR.I	1.52

(*Continued*)

USP4

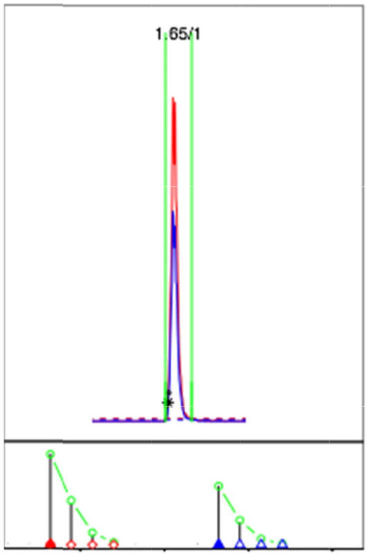

Peptide	L/H Ratio
K.EHLIDELDYVLVPTEAWNK.L	1.81
K.GEIAEAYAELIK.Q	1.93
K.IFQMDEGLNHIMPR.D	2.5
K.ILVVHLK.R	1.65
K.KFDLWSLPK.I	1.75
K.KKPYLELK.D	1.77
K.KPYLELKDANGR.P	20
K.LCENSDPTNVLSCHFSK.A	1.66
K.LFNIPAER.E	1.72
K.LLNWYGCVEGQQPIVR.K	2.17
K.LTLESLYQAVCDR.I	1.76
K.RLFTFSLVNSYGTADINSLAADGK.L	1.73
K.SRPSSTSSASALYGQPLLLSVPK.H	1.82
K.VEVYLLELK.L	1.54
K.VSVTFDPFCYLTLPLPLK.K	1.91
K.VSVTFDPFCYLTLPLPLKK.D	1.78
K.YVGFDSWDMYNVGEHNLFPGPIDNSGLFSDPE	1.86
R.DCIELFTTMETLGEHDPWYCPN.C	1
R.DKLDTVVEFPIR.G	1.8
R.GAQWYLIDSR.W	1.55
R.GLNMSEFVCNLSAR.P	2.77
R.KLFNIPAER.E	1.67
R.KVVEHGLFVK.H	1.64
R.LFTFSLVNSYGTADINSLAADGK.L	1.64
R.LSGIAAENMVVADVYNHR.F	1.56
R.NDSVIVDTFHGLFK.S	1.69
R.STLAMDWDSETRR.L	1.63
R.VMEVFLVPADPH.C	1
R.VTVPLMGAVSDLCEALSR.L	1.73

BAP1

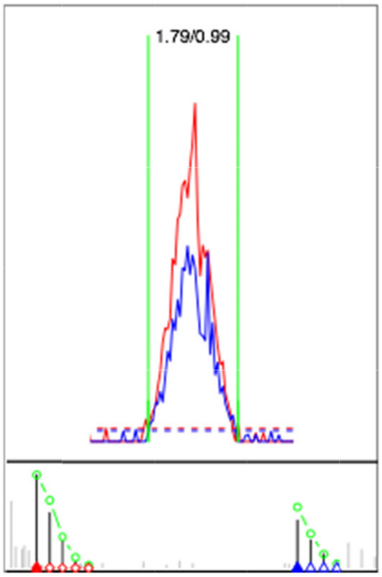

Peptide	L/H Ratio
R.QTVLEALQQLIR.V	1.97
R.DLGPVISTGLLHLAEDGVLSPLALTEGGK.G	1.83
R.FNLMAVVPDRR.I	1.79
K.CQGPVYGFIFLFK.W	1.76

(*Continued*)

USP14

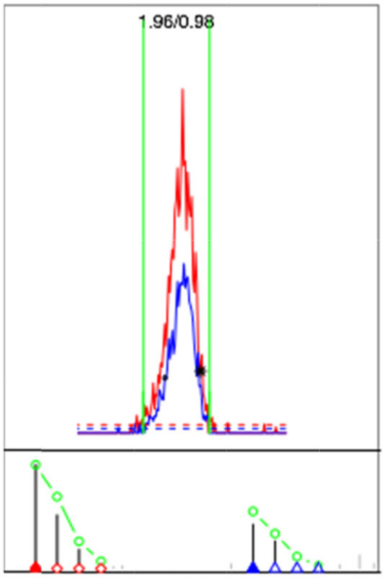

Peptide	L/H Ratio
H.IAYVLLYGPR.R	1.96
K.AQLFALTGVQPAR.Q	1.8
K.CTESEEEEVTKGKENQLQLSCFINQEVK.Y	2.12
K.EKFEGVELNTDEPPMVFK.A	2.1
K.ENQLQLSCFINQEVK.Y	1.94
K.FDDDKVSIVTPEDILR.L	2.01
K.FEGVELNTDEPPMVFK.A	1.88
K.FPLMLDMYELCTPELQEK.M	1.95
K.GEQGQYLQQDANECWIQMMR.V	2.35
K.GGTLKDDDWGNIK.I	1.87
K.GKENQLQLSCFINQEVK.Y	1.86
K.IKNGMTLLMMGSADALPEEPSAK.T	1.95
K.KVNQQPNTSDKK.S	1.97
K.LEAIEDDSVKETDSSSASAATPSK.K	2.07
K.NGMTLLMMGSADALPEEPSAK.T	1.84
K.RKQDEWIK.F	2.16
K.SLIDQFFGVEFETTMK.C	1.75
K.VSIVTPEDILR.L	1.95
K.YEPFSFADDIGSNNCGYYDLQAVLTHQGR.S	2.06
K.YLFTGLK.L	1.79
R.ASGEMASAQYITAALR.D	2.44
R.LPAYLTIQMVR.F	1.84
R.LSGGGDWHIAYVLLYGPR.R	2.16
R.RVEIMEEESEQ.-	1.87
R.SSSSGHYVSWVK.R	1.85
R.SSSSGHYVSWVKR.K	1.7
Y.DLQAVLTHQGR.S	1.66

OTUD4

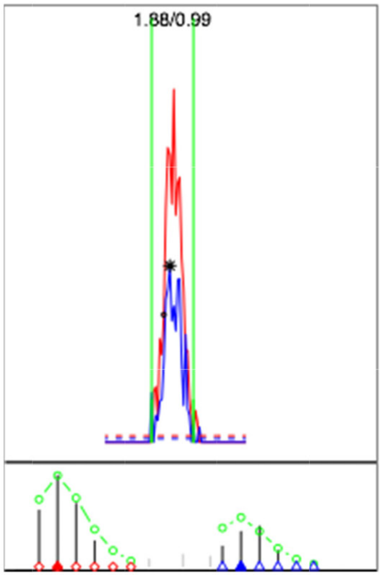

Peptide	L/H Ratio
K.ESSAMCQSLLYELLYEK.V	2.22
K.FLNADVQGIHSENGPVLVEELGK.K	1.93
R.FFFNLGVK.A	2.07
R.KADTALASIPPVAEGK.A	2.45
R.LENPQEWVGQVEISALSLMYR.K	1.88
R.MACIHYLR.E	1.84
R.QNIVLPSDEKGELDLSLENLDLSK.D	20

(*Continued*)

OTUD6B

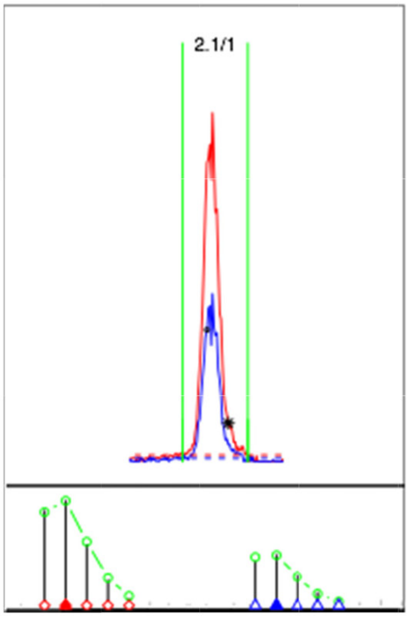

Peptide	L/H Ratio
K.ENKIDSVAVNISNLVLENQPPR.I	2.75
K.HREELEQLK.L	1.98
K.IDSVAVNISNLVLENQPPR.I	2.1
K.YCEDIVNTAAWGGQLELR.A	2.02
R.ALSHILQTPIEIIQADSPPIIVGEEYSK.K	2.21
R.HAYGLGEHYNSVTR.L	2.1
R.IAEAEIENLTGAR.H	2.09
R.SQTAEYMQSHVEDFLPFLTNPNTGDMYTPEEFQ	2.17

USP32

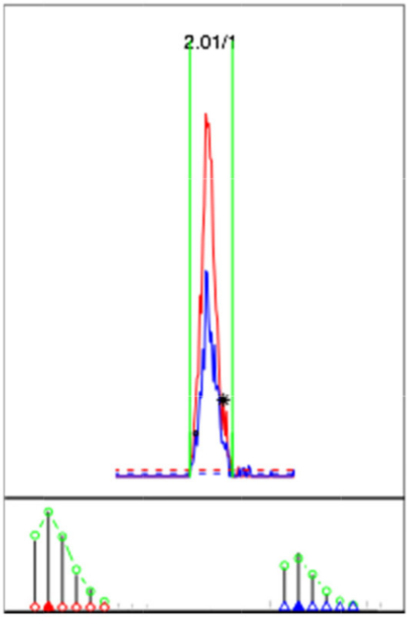

Peptide	L/H Ratio
K.IQDEQHLVIEVR.N	2.93
K.YSFGTAAHPMEQVEDR.I	2.61
R.ALYHWYGANLALPR.P	2.21
R.DMVVALLEVWK.D	2.1
R.DPALCQHKPLTPQGDELSEPR.I	2.47
R.FDLETFGPLVSPPIRPSLSEGLFNAFDENR.D	1.77
R.FNGFQQQDSQELLAFLLDGLHEDLNR.V	1.81
R.IGELSYEEALR.R	2.54
R.TDDIPELHMDLSDIVEGILNAHDTTK.M	2.2
R.TQQSNIWVNMGNVPSPNAPLKR.V	2.01

(*Continued*)

CYLD

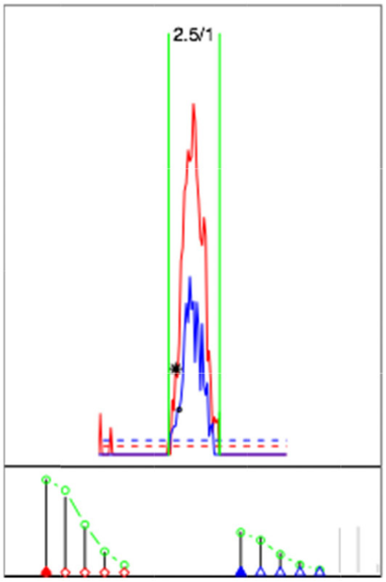

Peptide	L/H Ratio
K.FAEAPSCLIIQMPR.F	2.5
K.FTELLLAITNCEER.F	3.57
K.IFPSLELNITDLLEDTPR.Q	2.64
K.MSLEDLHSLDSR.R	6.57
K.VGVPTIQQLLEWSFINSNLK.F	2.81
K.VQDCYFYQIFMEK.N	3.07

USP12

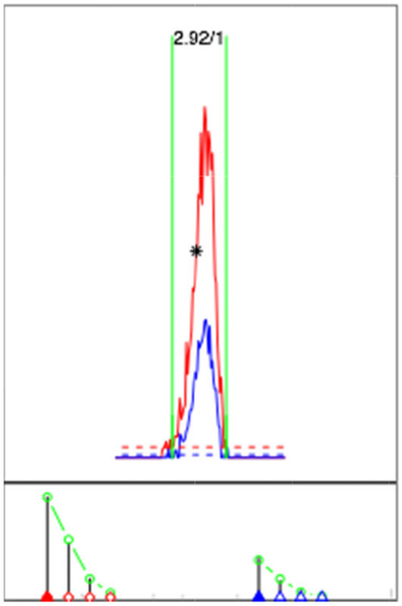

Peptide	L/H Ratio
R.VVFPLELR.L	2.92
R.LPNGNIDNENNNSTPDPTWVHEIFQGTLTNETR.	1

(*Continued*)

USP46

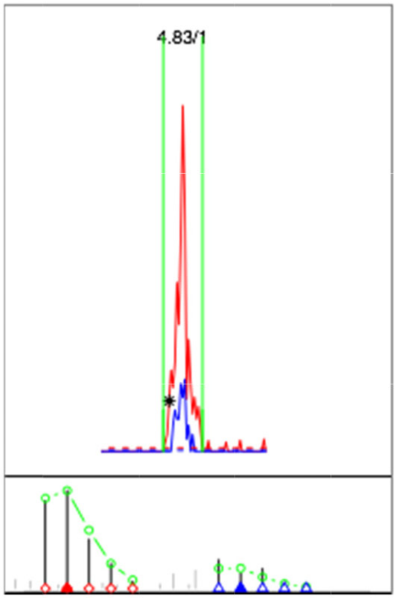

Peptide	L/H Ratio
R.NIASICNMGTNASALEK.D	20
K.IDAQAIEEFYGLTSDISK.N	4.83

UCH37

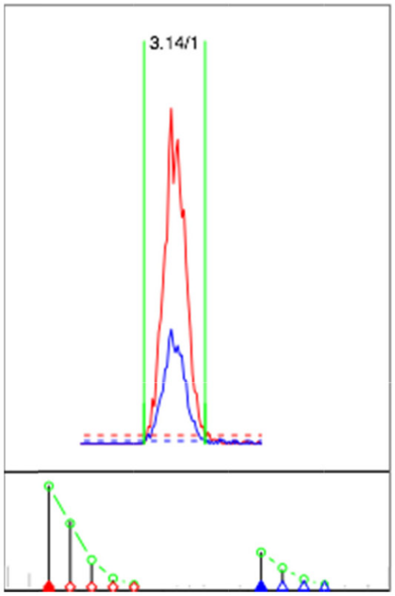

Peptide	L/H Ratio
-.MTGNAGEWCLMESDPGVFTELIK.G	3.48
K.EEDAFHFVSYVPVNGR.L	2.91
K.GLALSNSDVIR.Q	3.12
K.NQMLIEEEVQK.L	3.01
K.TLAEHQQLIPLVEK.A	3.39
K.TSAKEEDAFHFVSYVPVNGR.L	2.95
K.WQPGEEPAGSVVQDSR.L	4.34
M.TGNAGEWCLMESDPGVFTELIK.G	3.02
R.EGPIDLGACNQDDWISAVRPVIEK.R	3.48
R.EGPIDLGACNQDDWISAVRPVIEKR.I	0.64
R.FNLMAIVSDR.K	3.14
R.FNLMAIVSDRK.M	3.04
R.GAQVEEIWSLEPENFEK.L	3.05
R.LDTIFFAK.Q	3.09
R.LYELDGLR.E	3.11
R.QQMFEFDTK.T	3
R.YKIENIR.R	3.33

(*Continued*)

USP11

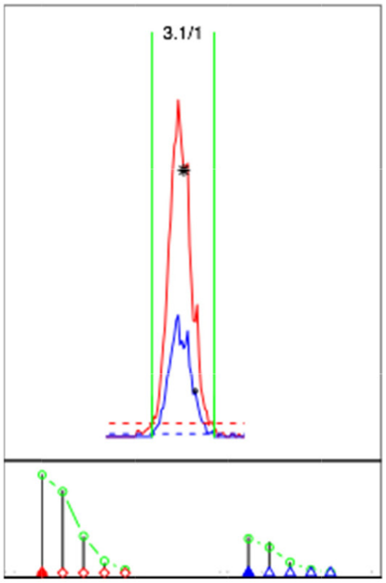

Peptide	L/H Ratio
K.EYVELCDAAGRPDQEVAQEAWQNHK.R	1.69
K.LYQLEEPLSSILDRDDIFVYEVSGR.I	3.19
K.QLFTLQTVNSNGTSDR.T	20
K.STLVCPDCGNVSVTFDPFCYLSVPLPISHK.R	20
K.VEVYPVELLLVR.H	3.1
K.VIELPNIQK.V	3.28
K.YDLIAVSNHYGGMR.D	3.27
R.CPFLLDNCLGTSQWPPR.R	20
R.DYNNSYYGLMLFGHPLLVSVPR.D	3.37
R.FTWEGLYNVLMYR.L	3.48
R.KVIELPNIQK.V	11.25
R.LQECIELFTTVETLEK.E	2.2
R.MMVADVFSHR.F	3.05
R.NDSVIVDTFHGLFK.S	1.69
R.SIVPHVEK.N	3.31
R.TTSPEEVHAQPYIAIDWEPEMK.K	3.64
R.VLEVFFIPMDPR.R	3.28

USP40

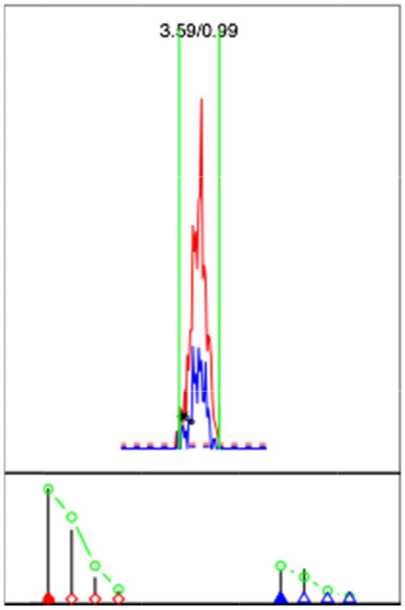

Peptide	L/H Ratio
K.IGISWNKK.Y	3.83
K.KIGISWNK.K	3.83
K.LPPFLTVSLLR.F	3.59
R.ILFSALETSLVGTSGHDLIYR.L	3.94
R.LYHGTIVNQIVCK.E	3.16
R.TYAPALDLVWNAAQGGTAGSLR.Q	3.97

(*Continued*)

USP10

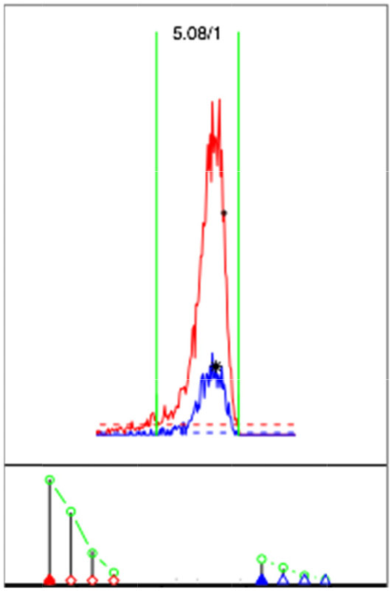

Peptide	L/H Ratio
K.ESATLQPFFTLQLDIQSDK.I	5.02
K.NIEYPVDLEISK.E	1.02
K.SWASLFHDSKPSSSSPVAYVETK.Y	6
K.TKQEVEISR.R	14.5
K.VINQYQVVKPTAER.T	5.64
K.VQRPCTSTPMIDSFVR.L	5.09
R.DIRPGAAFEPTYIYR.L	3.59
R.QADFVQTPITGIFGGHIR.S	4.92
R.QEDAEEYLGFILNGLHEEMLNLK.K	6.24
R.SSVELPPYSGTVLCGTQAVDKLPDGQEYQR.I	5.92
R.TAYLLYYR.R	4.02
R.TVQDALESLVAR.E	5.08

USP22

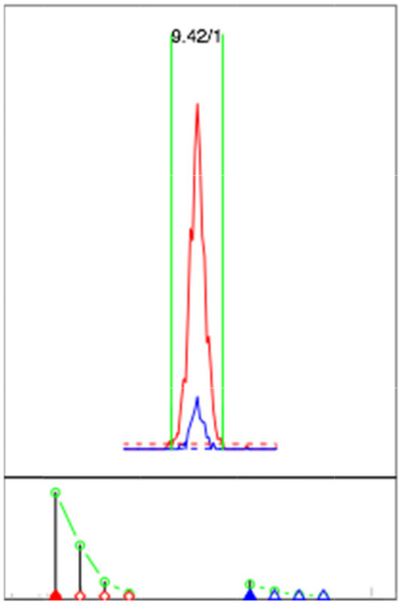

Peptide	L/H Ratio
K.ASIKDVLDSEGYLLFYHK.Q	11.59
K.DVLDSEGYLLFYHK.Q	20
K.FSTWEPTKR.E	8.58
K.LLHLVWTH.A	1
R.AIYQCFVWSGTAEAR.K	20
R.CEMQSPSSCLVCEMSSLFQEFYSGHR.S	10.42
R.FTRPEHLGSSAK.I	7.79
R.SPHIPYK.L	9.42

(*Continued*)

USP48

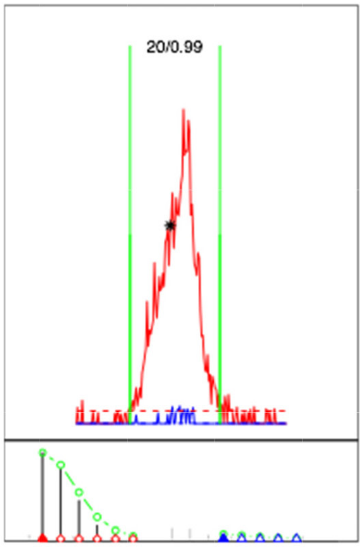

Peptide	L/H Ratio
K.ADEPIADYAAMDDVMQVCMPEEGFK.G	20
K.ALGLDTGQQQDAQEFSK.L	20
K.DPQSGEWYKFNDEDIEK.M	20
K.DYEPQTICEHLQYLFALLQNSNR.R	20
K.FEEWCIEMAEMR.K	20
K.FNDEDIEKMEGK.K	20
K.FYELELNIQGHK.Q	20
K.GGSYVYELSAVLIHR.G	20
K.GNPNCLVGIGEHIWLGEIDENSFHNIDDPNCER.	20
K.ILSDDCATLGTLGVIPESVILLK.A	20
K.IQIMHAFSVAPFDQNLSIDGK.I	20
K.ISHQNYIAYQK.Q	20
K.KLQLGIEEDLAEPSK.S	20
K.LFMSLLEDTLSK.Q	18.03
K.LIALIWPSEWQMIQK.L	20
K.LQLGIEEDLAEPSK.S	20
K.QLTDCISEFLKEEK.L	20
K.TSLPNLFQDK.N	20
K.WLDESTPTKPIDNHACLCSHDK.L	20
R.DNSKFEEWCIEMAEMR.K	20
R.EYTQATIYVHK.V	20
R.GVSAYSGHYIAHVK.D	20
R.ISEYAADIFYSR.Y	20
R.KEEEELNFNEDILCPHGELCISENER.R	20
R.LLSLPCTLNLQLMR.F	20
R.LPAGAEPYEFVSLEWLQK.W	20
R.LQTQEKPNTTVQVPAFLQELVDR.D	20
R.LQTQEKPNTTVQVPAFLQELVDRDNSK.F	20
R.NIVQQQFCGEYAYVTVCNQCGR.E	20
R.RYIDPSGFVK.A	17.31
R.WAETVRPEEVSQEHIETAYR.I	20

USP3

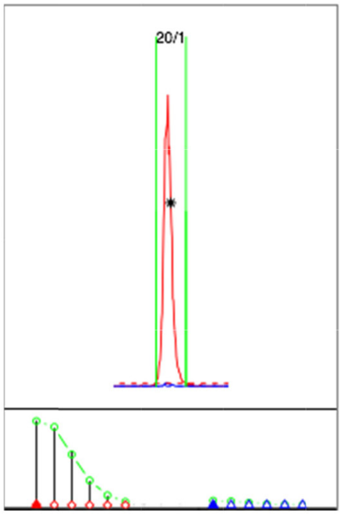

Peptide	L/H Ratio
K.AYILFYVEHQAK.A	20
K.CCINGASTVVTAIFGGILQNEVNCLICGTESR.K	20
K.FPNGSPSSWCCSVCR.S	20
K.SPWVCLTCSSVHCGR.Y	20
K.TLCALWQGSQTAFSPESLFYVVWK.I	20
K.VLCLHLK.R	20
K.VQHTVCMDCSSYSTYCYR.C	20
K.VREHLQNLENSAFTADR.H	20
N.AILQSLSNIEQFCCYFK.E	20
R.EHLQNLENSAFTADR.H	8.55
R.FHWTAYLR.N	20
R.KFDPFLDLSLDIPSQFR.S	20
R.NKVDTYVEFPLR.G	20
R.SFTDLEELDETELYMCHK.C	20

(*Continued*)

USP1

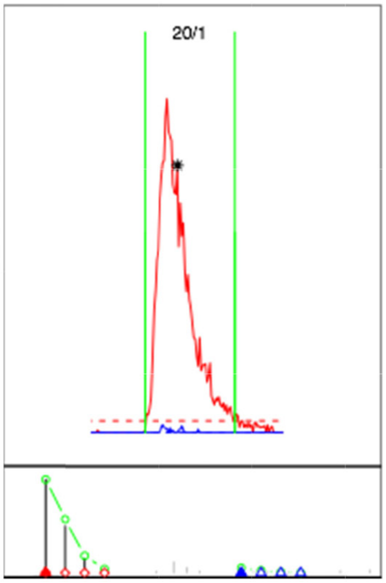

Peptide	L/H Ratio
K.GNFVVDQMCEIGKPEPLNEEEAR.G	20
K.HLFNIISR.K	20
K.LFQGQLVLR.T	20
K.MPEVITIHLK.C	20
K.NVAELPTKVEEIPHPK.E	20
K.NVEAIGLLGGQK.S	20
K.VTEEKDFLNSLSPSTSPTSTPYLLFYK.K	20
K.WLLFDDSEVK.V	20

USP16

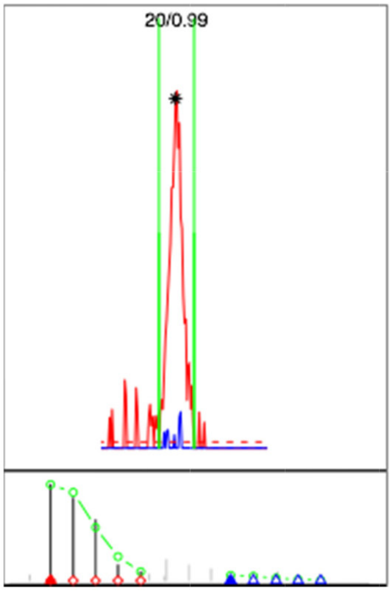

Peptide	L/H Ratio
K.AEEETEEKPSVWLCLK.C	20
K.AFGNSTEKLDEELK.N	20
K.ALVNVEWNICQDCK.T	20
K.DKAEEETEEKPSVWLCLK.C	19.13
K.FPEILDLAPFCTLK.C	19.26
K.GQWFHISDTHVQAVPTTK.V	20
K.NINMDNDLEVLTSSPTR.N	20
K.SNHISQEGVMHK.E	20
K.VLNSQAYLLFYER.I	20
R.EVFNTDECSIQHCLYQFTR.N	20
R.IFGGELTSMIMCDQCR.T	20
R.TANSHLSNLVLHGDIPQDFEMESK.G	20
R.TVSLVHESFLDLSLPVLDDQSGKK.S	20
R.VLYSLYGVVEHSGTMR.S	20

(*Continued*)

VCPIP1

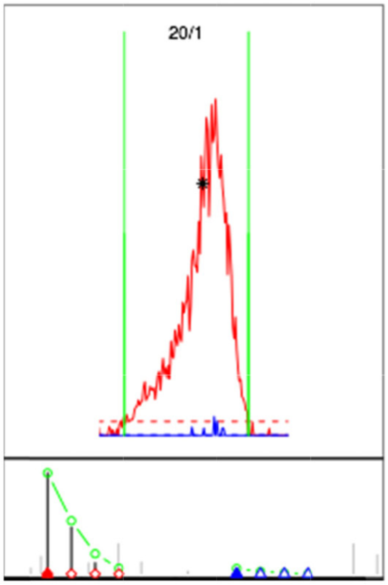

Peptide	L/H Ratio
K.CLLCGALSELHVPPEWLAPGGK.L	20
K.GHSLGTASGNPHLDPR.A	20
K.LPMNLLPK.A	20
K.SEQLHNVTAFQGK.G	1.11
K.SSTTEEELQESIAR.E	20
K.VRGDGSIVYLDGDR.T	20
R.AFLIEPEHVNTVGYGK.D	20
R.HEQQQLLGVEEVTDPDVVLHNLLR.N	20
R.LEEPASGSVSIECTECGQR.H	20
R.LVAAMEEVFMDK.H	20
R.NHYIPLVGIK.G	20
R.SGSLLYLHDTLEDIKR.A	20
